# Supplementary material for: Comparison of Three Common Intervertebral Disc Discectomies in the Treatment of Lumbar Disc Herniation: A Systematic Review and Meta-Analysis Based on Multiple Data
Source: J Clin Med. 2022 Nov 8;11(22):6604. doi: 10.3390/jcm11226604 (PMC9696245; doi:10.3390/jcm11226604)
Supplement: Supplementary file 1 [file jcm-11-06604-s001.zip › jcm-1927646-supplementary.pdf]

Supplementary files

Table S1 Search Strategy in Pubmed

Population（Patients with lumbar disc herniation）	＃1：((((lumbar disc herniation[Title/Abstract]) OR lumbar disc prolapse[Title/Abstract]) OR lumbar disc rupture[Title/Abstract]) OR lumbar intervertebral disc protrusion[Title/Abstract]) OR ldh[Title/Abstract]
Intervention（PTED）	＃2：((((((((percutaneous endoscopic lumbar discectomy[Title/Abstract]) OR percutaneous endoscopic discectomy[Title/Abstract]) OR minimally invasive transforaminal discectomy[Title/Abstract]) OR transforaminal endoscopic[Title/Abstract]) OR pted[Title/Abstract]) OR peld[Title/Abstract]) OR tessys[Title/Abstract]) OR yess[Title/Abstract]) OR peid[Title/Abstract]) OR percutaneous endoscopic interlaminar discectomy[Title/Abstract]
Comparision（MED and OD）	＃3：((((((microdiscectomy[Title/Abstract]) OR micro-endoscopy[Title/Abstract]) OR microscopic diskectomy[Title/Abstract]) OR microendoscopy discectomy[Title/Abstract]) OR discectom[Title/Abstract]) OR microsurgical enucleation discectomy[Title/Abstract]) OR med[Title/Abstract]<br>＃4：((((((((((((((((((fenestration discectomy[Title/Abstract]) OR open discectomy[Title/Abstract]) OR open lumbar discectomy[Title/Abstract]) OR standard discectomy[Title/Abstract]) OR traditional lumbar discectomy[Title/Abstract]) OR conventional lumbar discectomy[Title/Abstract]) OR vertebral plate resection[Title/Abstract]) OR hemilaminectomy[Title/Abstract]) OR semi-laminectomy[Title/Abstract]) OR disk excision from posterior approach[Title/Abstract]) OR vertebral plate decompression[Title/Abstract]) OR semi-lamina[Title/Abstract]) OR omni-posterior decompression[Title/Abstract]) OR omni-posterior resection[Title/Abstract]) OR small incision laminectomy[Title/Abstract]) OR laminectomy[Title/Abstract]) OR unilateral vertebral plate decompression[Title/Abstract]) OR transforaminal lumbar interbody fusion[Title/Abstract]) OR posterior lumbar interbody fusion[Title/Abstract]) OR tilf[Title/Abstract]) OR plif[Title/Abstract]
Study	＃5: ((((((((((((randomized controlled trial[Title/Abstract]) OR controlled clinical trial[Title/Abstract]) OR randomized[Title/Abstract]) OR review study[Title/Abstract]) OR retrospective studies[Title/Abstract]) OR retrospective analysis[Title/Abstract]) OR retrospective control study[Title/Abstract]) OR retrospective cohort study[Title/Abstract]) OR retrospective case control study[Title/Abstract]) OR prospective study[Title/Abstract]) OR control study[Title/Abstract]) OR comparative study[Title/Abstract]) OR clinical trial[Title/Abstract]) OR clinic trial[Title/Abstract]

＃1 AND＃2 AND (＃3 OR＃4) AND＃5

Supplementary Figure S1 Meta-analysis for surgical related indicators and postoperative curative effect

Study or Subgroup	Experimental Mean	Experimental SD	Experimental Total	Control Mean	Control SD	Control Total	Weight	Mean Difference IV, Random, 95% CI
Ariun et al 2015	93.89	32.33	36	46.9	14.74	50	7.1%	46.99 [35.67, 58.31]
Chen et al 2018	97.2	45.8	80	91.7	42.5	73	7.0%	5.50 [-8.49, 19.49]
Choi et al 2016	67	12	20	136.7	53	23	6.4%	-69.70 [-91.99, -47.41]
Hsu et al 2013	86.5	45.9	57	48.1	9.2	66	7.1%	38.40 [26.28, 50.52]
Jing et al 2021	49.94	13.7	31	61.68	11.93	31	7.3%	-11.74 [-18.13, -5.35]
Kim et al 2007	53	13	301	64.6	28.7	614	7.4%	-11.60 [-14.30, -8.90]
Lee et al 2009	45.8	11.1	25	73.8	25.7	29	7.2%	-28.00 [-38.32, -17.68]
Li et al 2018	65.83	32.24	48	69.2	18.98	30	7.1%	-3.37 [-14.74, 8.00]
Liu et al 2018	43	13	55	57	10	59	7.4%	-14.00 [-18.28, -9.72]
Mayer et al1993	40.7	11.3	20	58.2	15.2	20	7.2%	-17.50 [-25.80, -9.20]
Rajamani et al 2021	47.44	12.47	86	108.92	12.98	351	7.4%	-61.48 [-64.44, -58.52]
Song et al 2017	78.7	13.4	30	76.6	15.5	30	7.3%	2.10 [-5.23, 9.43]
Wang et al 2013	92.4	34.6	25	82.6	40.2	80	6.8%	9.80 [-6.37, 25.97]
Wang et al 2021	59.41	22.19	69	69.62	18.96	86	7.3%	-10.21 [-16.80, -3.62]
**Total (95% CI)**			**883**			**1542**	**100.0%**	**-8.74 [-24.15, 6.66]**

Heterogeneity: Tau² = 834.05; Chi² = 1111.75, df = 13 (P < 0.00001); I² = 99%
Test for overall effect: Z = 1.11 (P = 0.27)

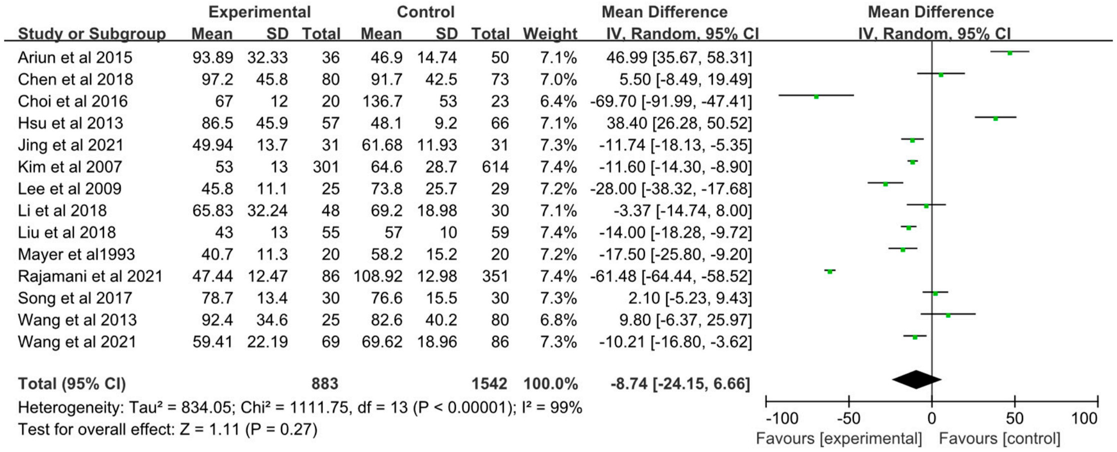

Figure S1a.Meta-analysis for duration of average operation time (min) between PTED and MED

Study or Subgroup	Experimental Mean	Experimental SD	Experimental Total	Control Mean	Control SD	Control Total	Weight	Mean Difference IV, Random, 95% CI
Ahn et al 2015	48.66	6.45	32	53.71	8.49	34	7.9%	-5.05 [-8.67, -1.43]
Ahn et al 2019	46.38	13.87	146	75.39	23.7	152	7.9%	-29.01 [-33.40, -24.62]
Chang et al 2017	75.03	20.21	60	77.95	18.56	50	7.8%	-2.92 [-10.17, 4.33]
Chen et al 2015	79.08	31.05	18	53.2	7.1	25	7.6%	25.88 [11.27, 40.49]
Ding et al 2017	74.4	39.6	50	66	22.8	50	7.7%	8.40 [-4.27, 21.07]
Jeoug et al 2006	78	36.71	22	110	29.68	25	7.3%	-32.00 [-51.25, -12.75]
Li et al 2018	71	19	33	121	22	30	7.8%	-50.00 [-60.20, -39.80]
Li et al 2015	82.7	13.2	30	57.5	9.1	26	7.9%	25.20 [19.32, 31.08]
Pan et al 2016	64.8	16.2	48	72	12.6	58	7.9%	-7.20 [-12.81, -1.59]
Rajamani et al 2021	47.44	12.47	86	130.5	32.81	145	7.9%	-83.06 [-89.02, -77.10]
Sun et al 2017	103.64	9.24	11	181.15	22.84	13	7.6%	-77.51 [-91.07, -63.95]
Wang et al 2013	92.4	34.6	25	80.9	31.4	16	7.2%	11.50 [-9.01, 32.01]
Yao et al 2016	63.38	20.25	47	140.09	57.07	58	7.5%	-76.71 [-92.50, -60.92]
**Total (95% CI)**			**608**			**682**	**100.0%**	**-22.50 [-41.03, -3.98]**

Heterogeneity: Tau² = 1124.80; Chi² = 996.25, df = 12 (P < 0.00001); I² = 99%
Test for overall effect: Z = 2.38 (P = 0.02)

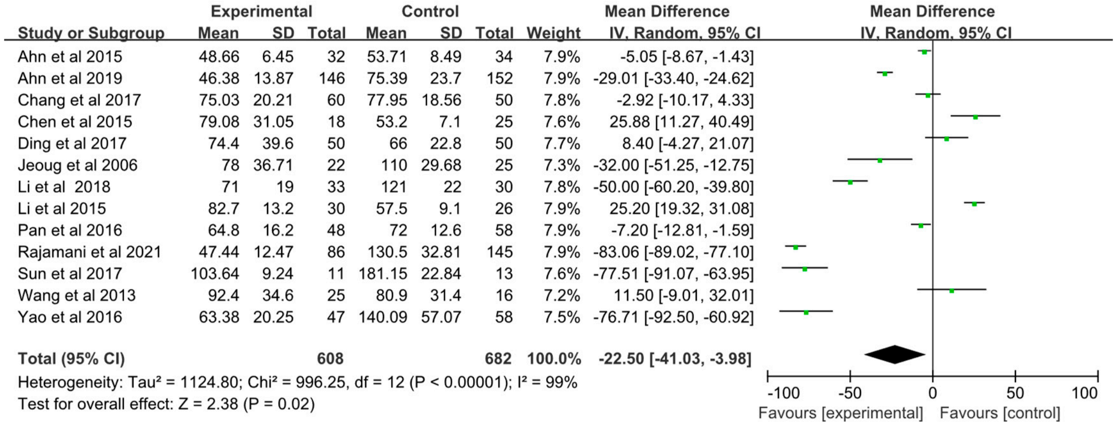

Figure S1b.Meta-analysis for duration of average operation time (min) between PTED and OD

Study or Subgroup	Experimental Mean	Experimental SD	Experimental Total	Control Mean	Control SD	Control Total	Weight	Mean Difference IV, Random, 95% CI
Jing et al 2021	20.93	2.59	31	30.63	2.57	31	15.9%	-9.70 [-10.98, -8.42]
Li et al 2018	19	18.01	48	29	23.47	30	13.5%	-10.00 [-19.82, -0.18]
Liu et al 2018	5	3	60	23	10	63	15.8%	-18.00 [-20.58, -15.42]
Rajamani et al 2021	24.5	9.26	86	61.58	9.46	351	15.8%	-37.08 [-39.27, -34.89]
Song et al 2017	50.6	18.3	30	100.7	45.6	30	10.1%	-50.10 [-67.68, -32.52]
Wang et al 2013	14.9	12.6	25	50.8	44.3	80	13.0%	-35.90 [-46.79, -25.01]
Wang et al 2021	7.54	2.15	69	30.47	7.01	86	15.9%	-22.93 [-24.50, -21.36]
**Total (95% CI)**			**349**			**671**	**100.0%**	**-24.96 [-34.12, -15.81]**

Heterogeneity: Tau² = 136.58; Chi² = 511.53, df = 6 (P < 0.00001); I² = 99%
Test for overall effect: Z = 5.34 (P < 0.00001)

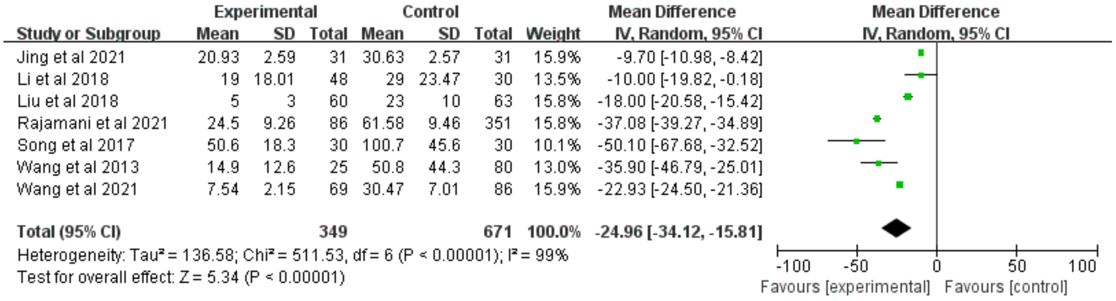

Figure S2a.Meta-analysis for duration of intraoperative blood loss (ml) between PTED and

MED

Study or Subgroup	Experimental Mean	Experimental SD	Experimental Total	Control Mean	Control SD	Control Total	Weight	Mean Difference IV, Random, 95% CI
Chang et al 2017	25.21	9.35	60	52.42	12.74	50	15.4%	-27.21 [-31.46, -22.96]
Li et al 2018	30	21	33	317	50	30	13.9%	-287.00 [-306.27, -267.73]
Li et al 2015	10.2	3.1	30	49.5	7.3	26	15.5%	-39.30 [-42.32, -36.28]
Pan et al 2014	8.35	2.99	10	99	22.33	10	14.6%	-90.65 [-104.61, -76.69]
Pan et al 2016	13.8	3.6	48	87.2	32.3	58	15.2%	-73.40 [-81.77, -65.03]
Rajamani et al 2021	24.5	9.26	86	56.4	5.13	145	15.5%	-31.90 [-34.03, -29.77]
Wang et al 2013	14.9	12.6	25	113.1	87.4	16	9.9%	-98.20 [-141.31, -55.09]
**Total (95% CI)**			**292**			**335**	**100.0%**	**-89.29 [-111.80, -66.79]**

Heterogeneity: Tau² = 850.09; Chi² = 827.98, df = 6 (P < 0.00001); I² = 99%
Test for overall effect: Z = 7.78 (P < 0.00001)

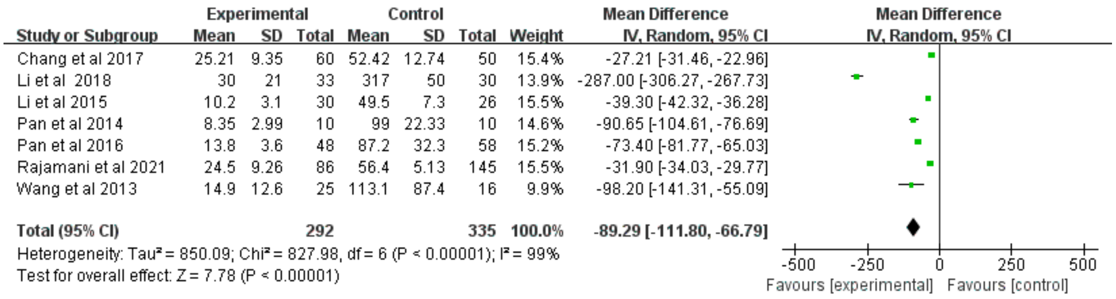

Figure S2b.Meta-analysis for duration of intraoperative blood loss (ml) between PTED and OD

Study or Subgroup	Experimental Mean	Experimental SD	Experimental Total	Control Mean	Control SD	Control Total	Weight	Mean Difference IV, Random, 95% CI
Jing et al 2021	0.73	0.18	31	1.8	0.19	31	25.1%	-1.07 [-1.16, -0.98]
Liu et al 2018	0.7	0.1	60	1.8	0.5	63	25.0%	-1.10 [-1.23, -0.97]
Rajamani et al 2021	0.8	0.15	86	3.98	1.2	351	25.0%	-3.18 [-3.31, -3.05]
Song et al 2017	0.8	0.2	30	1.7	0.6	30	24.8%	-0.90 [-1.13, -0.67]
**Total (95% CI)**			**207**			**475**	**100.0%**	**-1.56 [-2.63, -0.50]**

Heterogeneity: Tau² = 1.17; Chi² = 793.72, df = 3 (P < 0.00001); I² = 100%
Test for overall effect: Z = 2.89 (P = 0.004)

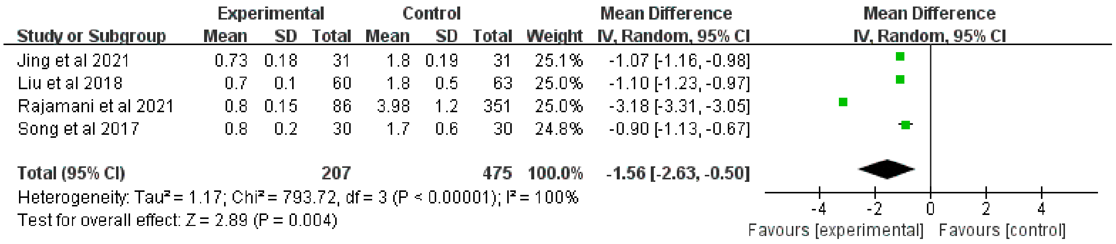

Figure S3a.Meta-analysis for size of incision(cm) between PTED and MED

Study or Subgroup	Experimental Mean	Experimental SD	Experimental Total	Control Mean	Control SD	Control Total	Weight	Mean Difference IV, Random, 95% CI
Chang et al 2017	2.1	0.08	60	5.15	0.31	50	20.8%	-3.05 [-3.14, -2.96]
Ding et al 2017	0.53	0.12	50	3.02	0.42	50	20.7%	-2.49 [-2.61, -2.37]
Pan et al 2014	0.51	0.02	10	4.9	1.29	10	17.8%	-4.39 [-5.19, -3.59]
Pan et al 2016	0.8	0	48	3.7	0.8	58		Not estimable
Rajamani et al 2021	0.8	0.15	86	5.7	1.98	145	20.2%	-4.90 [-5.22, -4.58]
Song et al 2017	0.8	0.2	30	1.7	0.6	30	20.5%	-0.90 [-1.13, -0.67]
**Total (95% CI)**			**284**			**343**	**100.0%**	**-3.11 [-3.99, -2.22]**

Heterogeneity: Tau² = 0.98; Chi² = 503.25, df = 4 (P < 0.00001); I² = 99%
Test for overall effect: Z = 6.87 (P < 0.00001)

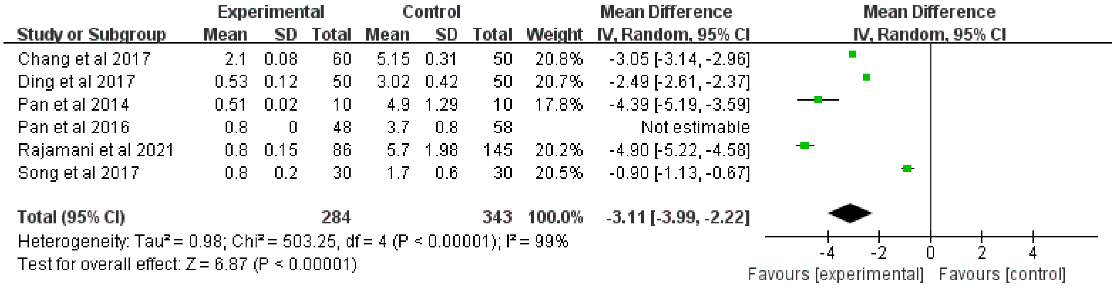

Figure S3b.Meta-analysis for size of incision(cm) between PTED and OD

Study or Subgroup	Experimental Mean	Experimental SD	Experimental Total	Control Mean	Control SD	Control Total	Weight	Mean Difference IV, Random, 95% CI
Chen et al 2016	1.36	1.14	80	2.94	1.62	73	49.5%	-1.58 [-2.03, -1.13]
Song et al 2017	1.3	0.5	30	4.6	0.7	30	50.5%	-3.30 [-3.61, -2.99]
**Total (95% CI)**			**110**			**103**	**100.0%**	**-2.45 [-4.13, -0.76]**

Heterogeneity: Tau² = 1.44; Chi² = 38.49, df = 1 (P < 0.00001); I² = 97%
Test for overall effect: Z = 2.85 (P = 0.004)

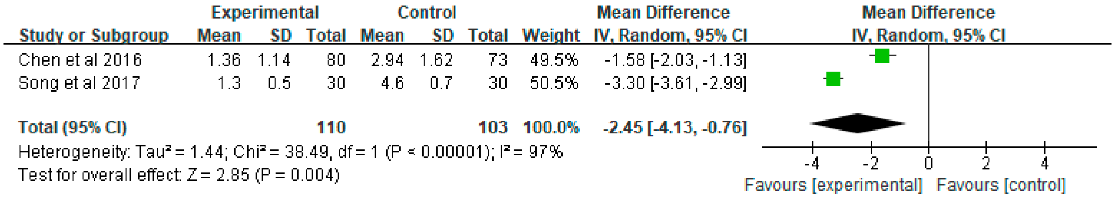

Figure S4a.Meta-analysis for postoperative bed rest time (day) between PTED and MED

Study or Subgroup	Experimental Mean	Experimental SD	Experimental Total	Control Mean	Control SD	Control Total	Weight	Mean Difference IV, Random, 95% CI
Ding et al 2017	1.02	0.34	50	5.99	0.73	50	38.0%	-4.97 [-5.19, -4.75]
Chang et al 2017	2.61	1.07	60	6.2	2.44	50	34.1%	-3.59 [-4.32, -2.86]
Sun et al 2017	1.23	0.41	11	8	2.27	13	27.9%	-6.77 [-8.03, -5.51]
**Total (95% CI)**			**121**			**113**	**100.0%**	**-5.00 [-6.27, -3.73]**

Heterogeneity: Tau^2 = 1.10; Chi^2 = 21.29, df = 2 (P < 0.0001); I^2 = 91%
Test for overall effect: Z = 7.71 (P < 0.00001)

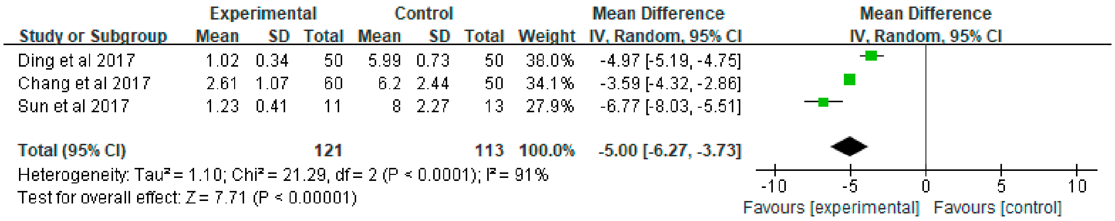

Figure S4b.Meta-analysis for postoperative bed rest time (day) between PTED and OD

Study or Subgroup	Experimental Mean	Experimental SD	Experimental Total	Control Mean	Control SD	Control Total	Weight	Mean Difference IV, Random, 95% CI
Ariun et al 2015	5.05	2.2	36	5.54	1.72	50	10.2%	-0.49 [-1.35, 0.37]
Chen et al 2018	8.1	4.2	80	11.2	3.8	73	8.9%	-3.10 [-4.37, -1.83]
Choi et al 2016	1.5	1.1	20	7.2	3.5	23	8.2%	-5.70 [-7.21, -4.19]
Jarebi et al 2021	2.55	1.785	29	3.61	1.624	29	10.1%	-1.06 [-1.94, -0.18]
Jing et al 2021	5.03	0.98	31	9.03	1.14	31	11.0%	-4.00 [-4.53, -3.47]
Lee et al 2009	0.9	0.5	25	3.8	1.4	29	11.0%	-2.90 [-3.45, -2.35]
Li et al 2018	3.78	1.83	48	4.8	1.57	30	10.5%	-1.02 [-1.78, -0.26]
Rajamani et al 2021	1.89	0.95	86	4.5	0.45	351	11.5%	-2.61 [-2.82, -2.40]
Wang et al 2013	7.8	3.2	25	11.2	5.9	80	7.2%	-3.40 [-5.20, -1.60]
Wang et al 2021	1.46	0.79	69	2.41	1.31	86	11.4%	-0.95 [-1.28, -0.62]
**Total (95% CI)**			**449**			**782**	**100.0%**	**-2.42 [-3.21, -1.63]**

Heterogeneity: Tau^2 = 1.41; Chi^2 = 173.59, df = 9 (P < 0.00001); I^2 = 95%
Test for overall effect: Z = 5.99 (P < 0.00001)

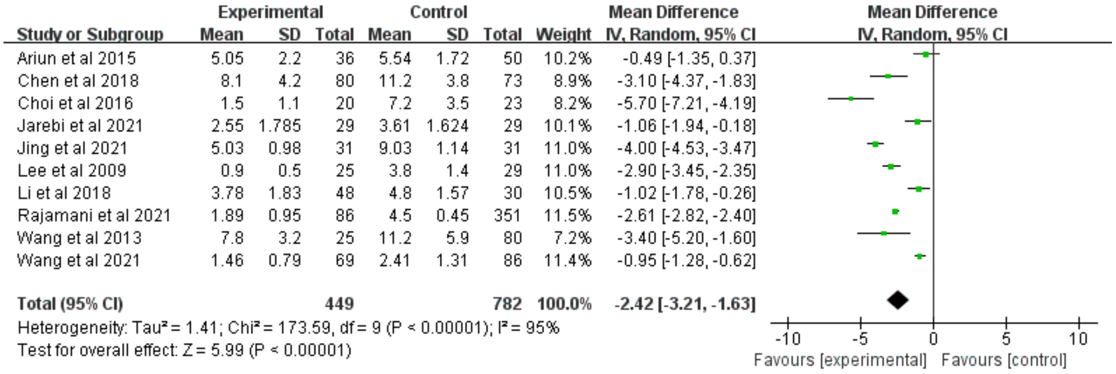

Figure S5a.Meta-analysis for hospitalization time between PTED and MED

Study or Subgroup	Experimental Mean	Experimental SD	Experimental Total	Control Mean	Control SD	Control Total	Weight	Mean Difference IV, Random, 95% CI
Ahn et al 2015	7.5	2.63	32	15.65	4.8	34	8.0%	-8.15 [-10.00, -6.30]
Ahn et al 2019	2.1	1.1	146	6.1	2.6	152	9.4%	-4.00 [-4.45, -3.55]
Chen et al 2015	1.89	0.74	18	12.28	7.58	25	6.4%	-10.39 [-13.38, -7.40]
Ding et al 2017	7.62	2.23	50	10.16	2.34	50	9.1%	-2.54 [-3.44, -1.64]
Li et al 2018	3.5	1.9	33	12.5	2.1	30	9.0%	-9.00 [-9.99, -8.01]
Li et al 2015	3.8	0.9	30	12.6	3.5	26	8.6%	-8.80 [-10.18, -7.42]
Pan et al 2014	1.9	0.74	10	5.6	1.26	10	9.1%	-3.70 [-4.61, -2.79]
Pan et al 2016	7.2	1.6	48	12.8	3.8	58	9.0%	-5.60 [-6.68, -4.52]
Rajamani et al 2021	1.89	0.95	86	6.5	2.1	145	9.5%	-4.61 [-5.01, -4.21]
Sun et al 2017	4.91	0.83	11	14.15	1.28	13	9.2%	-9.24 [-10.09, -8.39]
Wang et al 2013	7.8	3.2	25	16.9	9.8	16	4.0%	-9.10 [-14.06, -4.14]
Yao et al 2016	8.13	2.91	47	12.74	4.04	58	8.7%	-4.61 [-5.94, -3.28]
**Total (95% CI)**			**536**			**617**	**100.0%**	**-6.38 [-7.69, -5.08]**

Heterogeneity: Tau^2 = 4.66; Chi^2 = 273.27, df = 11 (P < 0.00001); I^2 = 96%
Test for overall effect: Z = 9.57 (P < 0.00001)

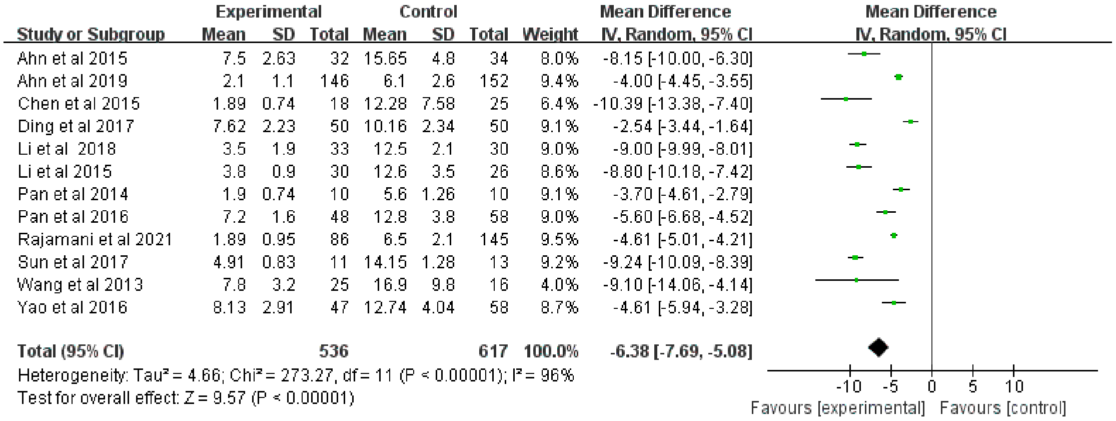

Figure S5b.Meta-analysis for hospitalization time between PTED and OD

Study or Subgroup	Experimental Mean	Experimental SD	Experimental Total	Control Mean	Control SD	Control Total	Weight	Mean Difference IV, Random, 95% CI
Chen et al 2018	0.6	1.4	74	0.4	1	63	8.5%	0.20 [-0.20, 0.60]
Chen et al 2020	0.5	1.3	119	0.4	0.9	122	13.1%	0.10 [-0.18, 0.38]
Choi et al 2016	1.7	1.2	20	2.3	0.8	23	4.4%	-0.60 [-1.22, 0.02]
Gibson et al 2016	1.9	2.6	58	3.5	3.1	65	1.9%	-1.60 [-2.61, -0.59]
Jarebi et al 2021	2.24	2.65	29	1.75	2.47	29	1.1%	0.49 [-0.83, 1.81]
Li et al 2018	0.61	1.16	48	0.73	1.1	30	6.0%	-0.12 [-0.63, 0.39]
Liu et al 2018	1.2	0.5	55	1.1	0.3	59	20.6%	0.10 [-0.05, 0.25]
Meyer et al 2020	2.1	2.9	23	3	3.6	24	0.6%	-0.90 [-2.77, 0.97]
Pan et al 2016	2.9	2.5	25	3.5	3.1	29	0.9%	-0.60 [-2.09, 0.89]
Rajamani et al 2021	0.73	0.45	86	0.72	0.21	351	24.0%	0.01 [-0.09, 0.11]
Wang et al 2021	0.56	0.5	69	0.68	0.62	86	19.1%	-0.12 [-0.30, 0.06]
**Total (95% CI)**			**606**			**881**	**100.0%**	**-0.04 [-0.18, 0.10]**

Heterogeneity: Tau^2 = 0.02; Chi^2 = 20.43, df = 10 (P = 0.03); I^2 = 51%
Test for overall effect: Z = 0.52 (P = 0.60)

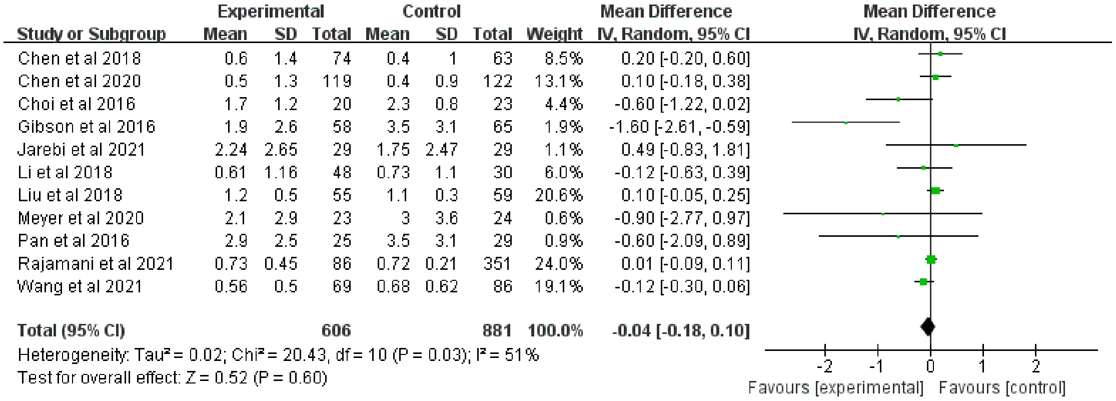

Figure S6a.Meta-analysis for VAS score of legs between PTED and MED

Study or Subgroup	Experimental Mean	Experimental SD	Experimental Total	Control Mean	Control SD	Control Total	Weight	Mean Difference IV, Fixed, 95% CI
Ahn et al 2015	2.06	0.84	32	2.32	1.01	34	3.3%	-0.26 [-0.71, 0.19]
Ahn et al 2019	1.44	1.02	146	1.32	1.02	152	12.3%	0.12 [-0.11, 0.35]
Liu et al 2016	1.1	0.8	209	1.2	0.7	192	30.6%	-0.10 [-0.25, 0.05]
Pan et al 2016	0.9	0.3	48	1	0.9	58	10.9%	-0.10 [-0.35, 0.15]
Rajamani et al 2021	0.73	0.45	86	0.81	0.62	145	34.4%	-0.08 [-0.22, 0.06]
Yao et al 2016	1.83	0.761	47	1.621	0.679	58	8.5%	0.21 [-0.07, 0.49]
**Total (95% CI)**			**568**			**639**	**100.0%**	**-0.05 [-0.13, 0.04]**

Heterogeneity: Chi² = 6.99, df = 5 (P = 0.22); I² = 29%
Test for overall effect: Z = 1.09 (P = 0.28)

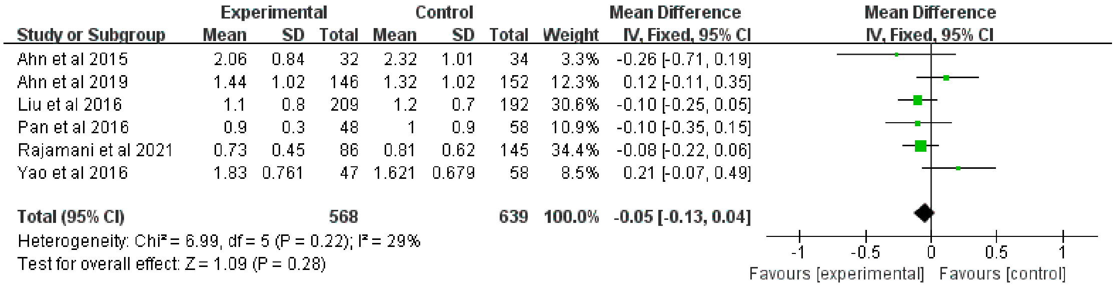

Figure S6b.Meta-analysis for VAS score of legs between PTED and OD

Study or Subgroup	Experimental Mean	Experimental SD	Experimental Total	Control Mean	Control SD	Control Total	Weight	Mean Difference IV, Random, 95% CI
Chen et al 2018	0.5	1.3	74	0.4	0.8	63	12.6%	0.10 [-0.26, 0.46]
Chen et al 2020	0.5	1.2	119	0.4	0.8	122	13.5%	0.10 [-0.16, 0.36]
Choi et al 2016	2	0.6	20	3.7	1	23	11.3%	-1.70 [-2.19, -1.21]
Gibson et al 2016	2.5	2.5	58	3	2.8	55	6.8%	-0.50 [-1.48, 0.48]
Lee et al 2009	2.9	2.4	25	3.1	2.5	29	4.8%	-0.20 [-1.51, 1.11]
Li et al 2018	0.65	0.88	48	1.87	1.46	30	10.4%	-1.22 [-1.80, -0.64]
Liu et al 2018	0.9	0.7	55	1.6	0.4	59	13.8%	-0.70 [-0.91, -0.49]
Rajamani et al 2021	1.58	0.52	86	1.67	0.56	351	14.3%	-0.09 [-0.21, 0.03]
Wang et al 2021	0.41	0.5	69	0.74	1.62	86	12.6%	-0.33 [-0.69, 0.03]
**Total (95% CI)**			**554**			**818**	**100.0%**	**-0.49 [-0.84, -0.14]**

Heterogeneity: Tau² = 0.22; Chi² = 79.89, df = 8 (P < 0.00001); I² = 90%
Test for overall effect: Z = 2.75 (P = 0.006)

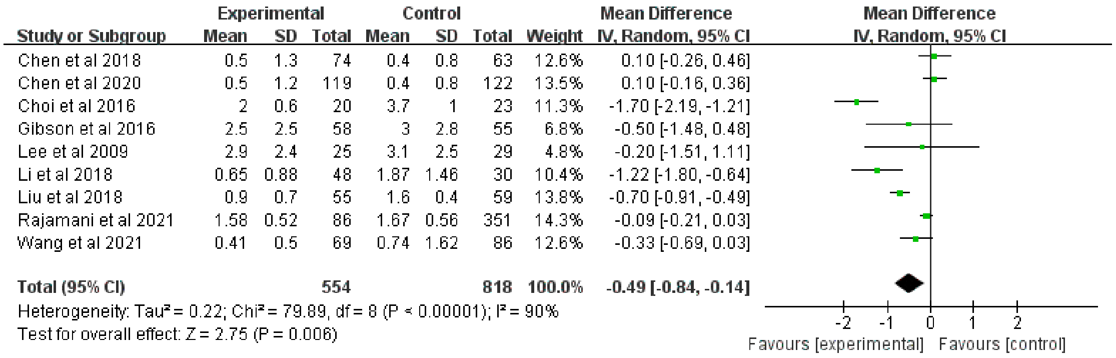

Figure S7a.Meta-analysis for VAS score of the low back between PTED and MED

Study or Subgroup	Experimental Mean	Experimental SD	Experimental Total	Control Mean	Control SD	Control Total	Weight	Mean Difference IV, Random, 95% CI
Ahn et al 2015	2.5	0.62	32	2.91	0.67	34	16.4%	-0.41 [-0.72, -0.10]
Ahn et al 2019	1.91	1.01	146	1.76	0.77	152	16.8%	0.15 [-0.05, 0.35]
Liu et al 2016	3.1	1.2	209	1.4	0.8	192	16.8%	1.70 [1.50, 1.90]
Pan et al 2016	1.6	0.4	48	1.4	0.2	58	17.1%	0.20 [0.08, 0.32]
Rajamani et al 2021	1.58	0.52	86	1.74	0.97	145	16.9%	-0.16 [-0.35, 0.03]
Yao et al 2016	2.468	1.039	47	2.155	0.894	58	16.0%	0.31 [-0.06, 0.69]
**Total (95% CI)**			**568**			**639**	**100.0%**	**0.30 [-0.28, 0.88]**

Heterogeneity: Tau² = 0.51; Chi² = 237.38, df = 5 (P < 0.00001); I² = 98%
Test for overall effect: Z = 1.02 (P = 0.31)

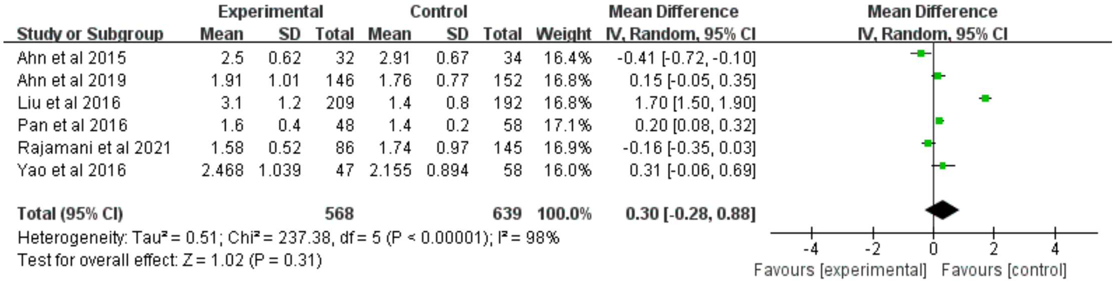

Figure S7b.Meta-analysis for VAS score of the low back between PTED and OD

Study or Subgroup	Experimental Mean	Experimental SD	Experimental Total	Control Mean	Control SD	Control Total	Weight	Mean Difference IV, Random, 95% CI
Ding et al 2017	0.41	0.23	50	0.45	0.21	50	22.3%	-0.04 [-0.13, 0.05]
Chang et al 2017	2.21	1.57	60	4.82	2.31	50	19.3%	-2.61 [-3.36, -1.86]
Li et al 2018	2	1.3	33	2.1	1.8	30	19.1%	-0.10 [-0.88, 0.68]
Pan et al 2014	1.8	0.79	10	1.9	0.74	10	19.9%	-0.10 [-0.77, 0.57]
Sun et al 2017	0.73	0.47	11	2.08	1.26	13	19.4%	-1.35 [-2.09, -0.61]
**Total (95% CI)**			**164**			**153**	**100.0%**	**-0.81 [-1.71, 0.08]**

Heterogeneity: Tau² = 0.93; Chi² = 55.42, df = 4 (P < 0.00001); I² = 93%
Test for overall effect: Z = 1.78 (P = 0.07)

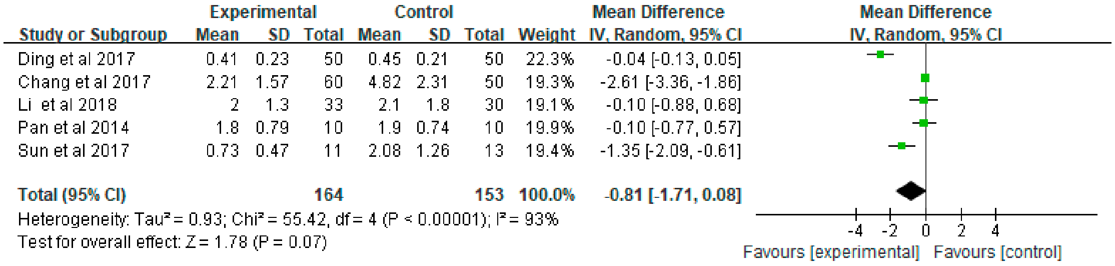

Figure S8. Meta-analysis for postoperative comprehensive VAS score between PTED and OD

Study or Subgroup	Experimental Mean	Experimental SD	Experimental Total	Control Mean	Control SD	Control Total	Weight	Mean Difference IV, Random, 95% CI
Ariun et al 2015	3.61	8.5	36	4.96	10.34	50	7.6%	-1.35 [-5.34, 2.64]
Chen et al 2018	3.9	7.6	74	3.2	5.7	63	9.6%	0.70 [-1.53, 2.93]
Chen et al 2020	4.1	8.7	119	3.9	6.5	122	9.9%	0.20 [-1.74, 2.14]
Choi et al 2016	12.5	7.5	20	20.2	7.2	23	7.1%	-7.70 [-12.11, -3.29]
Gibson et al 2016	18	17	58	22	20	65	4.9%	-4.00 [-10.54, 2.54]
Hsu et al 2013	6.42	9.82	46	3.29	6.94	55	8.3%	3.13 [-0.25, 6.51]
Jarebi et al 2021	21.1	16.3	29	23.5	16.5	29	3.6%	-2.40 [-10.84, 6.04]
Lee et al 2009	20.7	15.9	25	18.2	15.4	29	3.7%	2.50 [-5.88, 10.88]
Li et al 2018	6.76	13.07	48	8	8.8	30	6.6%	-1.24 [-6.10, 3.62]
Liu et al 2018	19	6	55	24	11	59	8.5%	-5.00 [-8.22, -1.78]
Rajamani et al 2021	17.8	9.72	86	21.92	9.32	351	9.6%	-4.12 [-6.39, -1.85]
Song et al 2017	1.3	0.4	30	1.2	0.3	30	11.0%	0.10 [-0.08, 0.28]
Wang et al 2021	31.23	6.59	69	39.85	7.81	86	9.6%	-8.62 [-10.89, -6.35]
**Total (95% CI)**			**695**			**992**	**100.0%**	**-2.21 [-4.17, -0.25]**

Heterogeneity: Tau² = 9.05; Chi² = 96.75, df = 12 (P < 0.00001); I² = 88%
Test for overall effect: Z = 2.21 (P = 0.03)

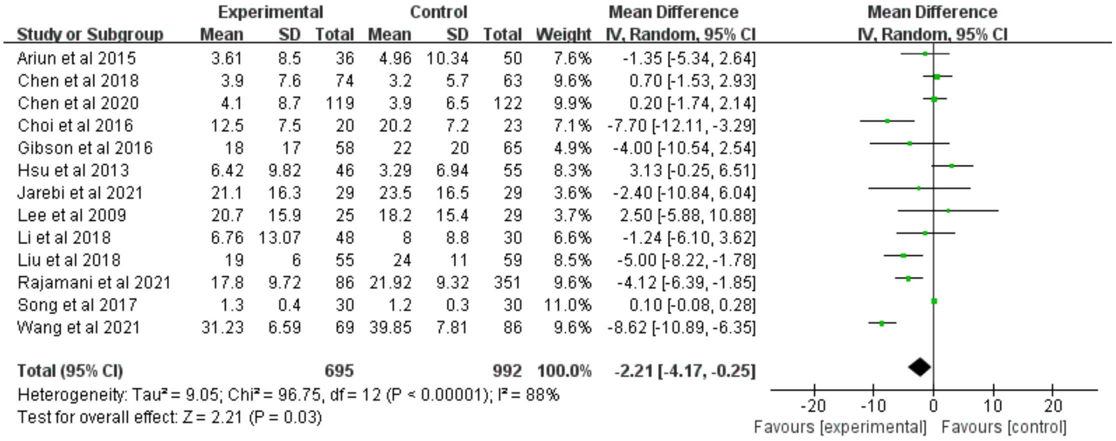

Figure S9a.Meta-analysis for postoperative ODI index between PTED and MED

Study or Subgroup	Experimental Mean	Experimental SD	Experimental Total	Control Mean	Control SD	Control Total	Weight	Mean Difference IV, Random, 95% CI
Ahn et al 2015	9.63	2.31	32	10.68	2.67	34	11.2%	-1.05 [-2.25, 0.15]
Ahn et al 2019	13.88	12.16	146	14	11.06	152	4.4%	-0.12 [-2.76, 2.52]
Ding et al 2017	2.55	2.04	50	2.65	2.15	50	14.4%	-0.10 [-0.92, 0.72]
Li et al 2015	2.1	1	30	2.1	2	26	14.1%	0.00 [-0.85, 0.85]
Liu et al 2016	11.4	3.2	209	12.4	3.6	192	15.6%	-1.00 [-1.67, -0.33]
Pan et al 2016	8.6	2.9	48	10.1	1.5	58	13.6%	-1.50 [-2.41, -0.59]
Rajamani et al 2021	10.62	2.83	86	12.05	6.32	145	11.3%	-1.43 [-2.62, -0.24]
Sun et al 2017	10.91	6.02	11	18.62	11.18	13	0.8%	-7.71 [-14.75, -0.67]
Yao et al 2016	12.511	2.339	47	11.828	1.687	58	14.6%	0.68 [-0.11, 1.48]
**Total (95% CI)**			**659**			**728**	**100.0%**	**-0.62 [-1.25, 0.01]**

Heterogeneity: Tau² = 0.55; Chi² = 24.74, df = 8 (P = 0.002); I² = 68%
Test for overall effect: Z = 1.93 (P = 0.05)

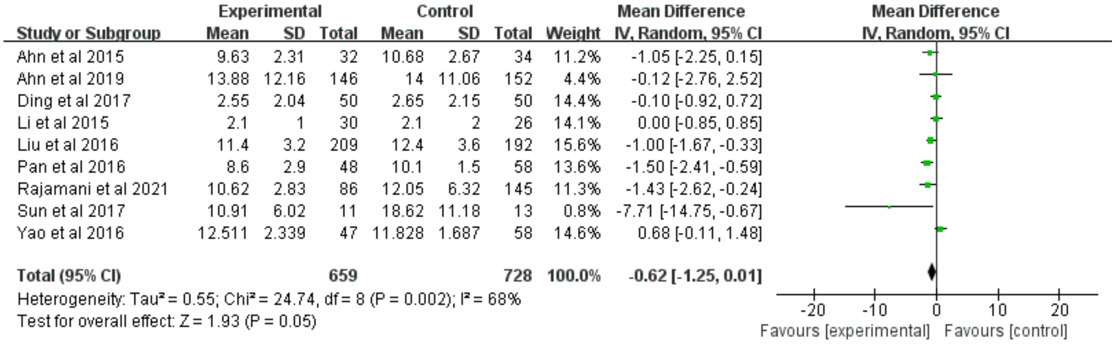

Figure S9b.Meta-analysis for postoperative ODI index between PTED and OD

Study or Subgroup	Experimental Events	Experimental Total	Control Events	Control Total	Weight	Odds Ratio M-H, Fixed, 95% CI
Chen et al 2018	6	80	9	73	12.6%	0.58 [0.19, 1.71]
Chen et al 2020	16	119	19	122	23.4%	0.84 [0.41, 1.73]
Gibson et al 2016	6	70	1	70	1.3%	6.47 [0.76, 55.21]
Hsu et al 2013	2	57	1	66	1.3%	2.36 [0.21, 26.77]
Jarebi et al 2021	3	29	2	29	2.6%	1.56 [0.24, 10.09]
Jing et al 2021	8	31	8	31	8.6%	1.00 [0.32, 3.12]
Kim et al 2007	9	295	10	607	9.1%	1.88 [0.76, 4.67]
Lee et al 2009	1	25	3	29	3.8%	0.36 [0.04, 3.71]
Liu et al 2018	2	60	4	63	5.4%	0.51 [0.09, 2.89]
Mayer et al1993	0	20	1	20	2.1%	0.32 [0.01, 8.26]
Meyer et al 2020	0	23	1	24	2.1%	0.33 [0.01, 8.61]
Rajamani et al 2021	10	86	38	238	25.7%	0.69 [0.33, 1.46]
Wang et al 2013	1	25	3	80	2.0%	1.07 [0.11, 10.76]
**Total (95% CI)**		**920**		**1452**	**100.0%**	**0.94 [0.67, 1.32]**
Total events	64		100			

Heterogeneity: Chi² = 9.64, df = 12 (P = 0.65); I² = 0%
Test for overall effect: Z = 0.38 (P = 0.71)

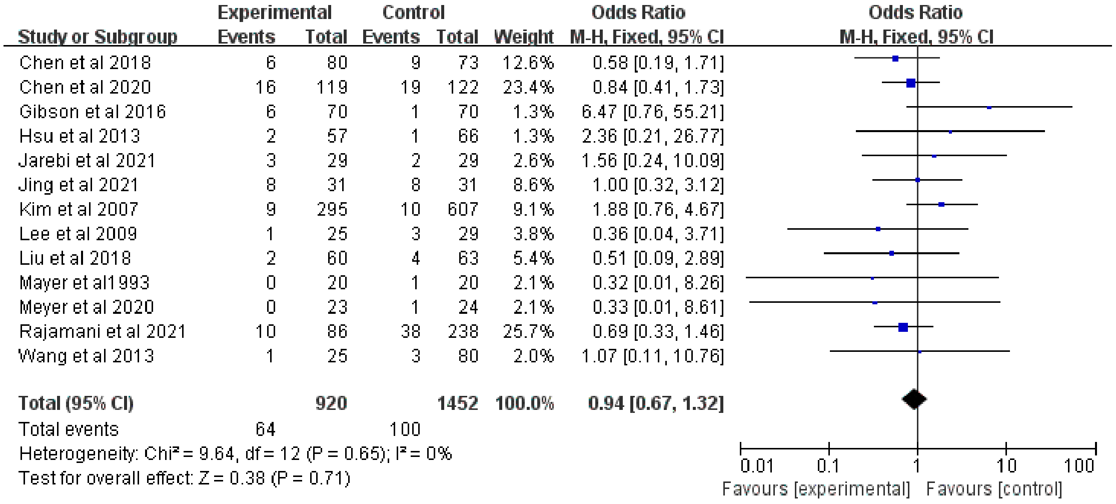

Figure S10a.Meta-analysis for incidence of complications between PTED and MED

Study or Subgroup	Experimental Events	Experimental Total	Control Events	Control Total	Weight	Odds Ratio M-H, Fixed, 95% CI
Ahn et al 2015	4	32	4	18	12.3%	0.50 [0.11, 2.30]
Chen et al 2015	0	18	3	25	7.9%	0.17 [0.01, 3.58]
Ding et al 2017	1	50	3	50	8.1%	0.32 [0.03, 3.18]
Hsu et al 2013	3	48	4	18	15.0%	0.23 [0.05, 1.17]
Li et al 2018	1	33	0	30	1.4%	2.82 [0.11, 71.78]
Liu et al 2016	11	209	12	192	32.6%	0.83 [0.36, 1.94]
Ma et al 2022	8	46	7	45	16.1%	1.14 [0.38, 3.47]
Pan et al 2014	1	10	0	10	1.2%	3.32 [0.12, 91.60]
Wang et al 2013	1	25	1	16	3.2%	0.63 [0.04, 10.76]
Yao et al 2016	4	47	1	58	2.3%	5.30 [0.57, 49.15]
**Total (95% CI)**		**518**		**462**	**100.0%**	**0.81 [0.50, 1.31]**
Total events	34		35			

Heterogeneity: Chi² = 8.69, df = 9 (P = 0.47); I² = 0%
Test for overall effect: Z = 0.86 (P = 0.39)

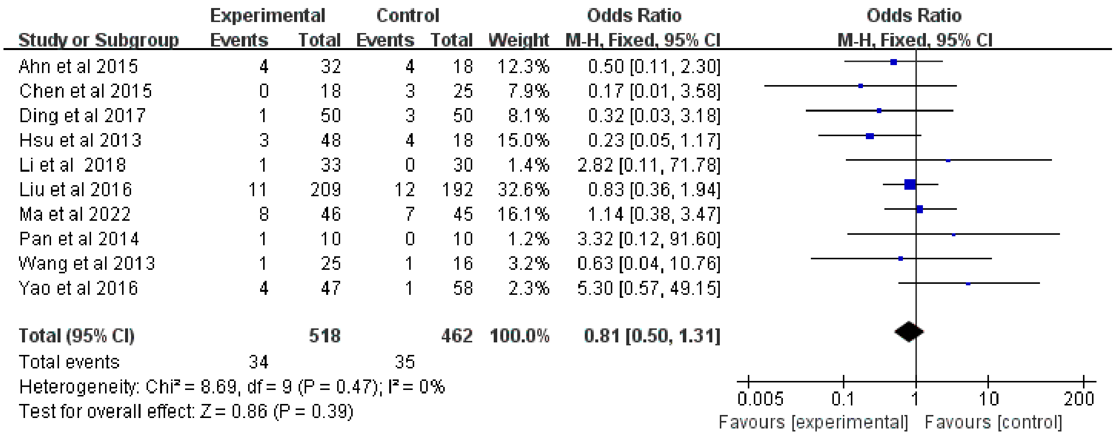

Figure S10b.Meta-analysis for incidence of complications between PTED and OD

Study or Subgroup	Experimental Events	Experimental Total	Control Events	Control Total	Weight	Odds Ratio M-H, Fixed, 95% CI
Chen et al 2018	5	80	3	73	6.8%	1.56 [0.36, 6.75]
Chen et al 2020	10	119	5	122	10.4%	2.15 [0.71, 6.48]
Choi et al 2016	1	20	0	23	1.0%	3.62 [0.14, 93.84]
Hsu et al 2013	2	57	0	66	1.0%	5.99 [0.28, 127.42]
Jarebi et al 2021	4	29	3	29	5.9%	1.39 [0.28, 6.83]
Jing et al 2021	2	31	1	31	2.2%	2.07 [0.18, 24.07]
Kim et al 2007	14	295	26	607	37.2%	1.11 [0.57, 2.17]
Lee et al 2009	1	25	3	29	6.1%	0.36 [0.04, 3.71]
Li et al 2018	1	48	1	30	2.8%	0.62 [0.04, 10.25]
Liu et al 2018	3	60	2	63	4.3%	1.61 [0.26, 9.96]
Mayer et al1993	1	20	0	20	1.1%	3.15 [0.12, 82.16]
Meyer et al 2020	1	23	2	24	4.3%	0.50 [0.04, 5.92]
Rajamani et al 2021	8	86	17	351	14.0%	2.02 [0.84, 4.84]
Wang et al 2013	1	25	1	80	1.1%	3.29 [0.20, 54.63]
Wang et al 2021	4	69	1	86	1.9%	5.23 [0.57, 47.92]
**Total (95% CI)**		**987**		**1634**	**100.0%**	**1.55 [1.07, 2.24]**
Total events	58		65			

Heterogeneity: Chi² = 7.05, df = 14 (P = 0.93); I² = 0%
Test for overall effect: Z = 2.30 (P = 0.02)

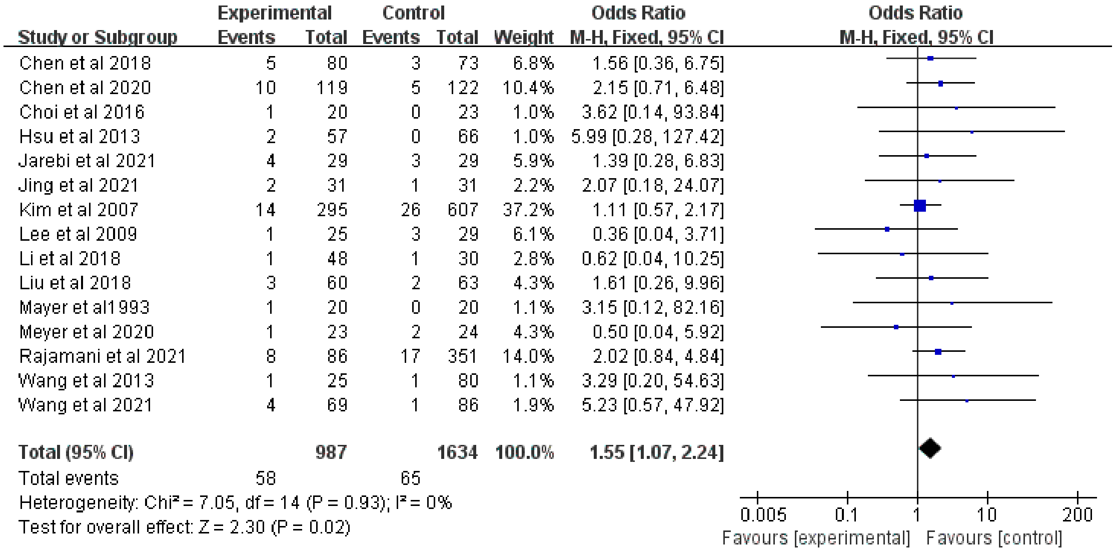

Figure S11a.Meta-analysis for incidence of recurrence between PTED and MED

Study or Subgroup	Experimental Events	Experimental Total	Control Events	Control Total	Weight	Odds Ratio M-H, Fixed, 95% CI
Ahn et al 2015	1	32	1	34	4.3%	1.06 [0.06, 17.77]
Ahn et al 2019	6	146	5	152	21.5%	1.26 [0.38, 4.22]
Liu et al 2016	12	209	0	192	2.2%	24.37 [1.43, 414.43]
Ma et al 2022	3	46	2	45	8.7%	1.50 [0.24, 9.43]
Rajamani et al 2021	8	86	19	145	58.8%	0.68 [0.28, 1.63]
Wang et al 2013	1	25	0	16	2.6%	2.02 [0.08, 52.68]
Yao et al 2016	5	47	0	58	1.8%	15.14 [0.82, 281.27]
**Total (95% CI)**		**591**		**642**	**100.0%**	**1.72 [1.02, 2.90]**
Total events	36		27			

Heterogeneity: Chi² = 10.24, df = 6 (P = 0.11); I² = 41%
Test for overall effect: Z = 2.05 (P = 0.04)

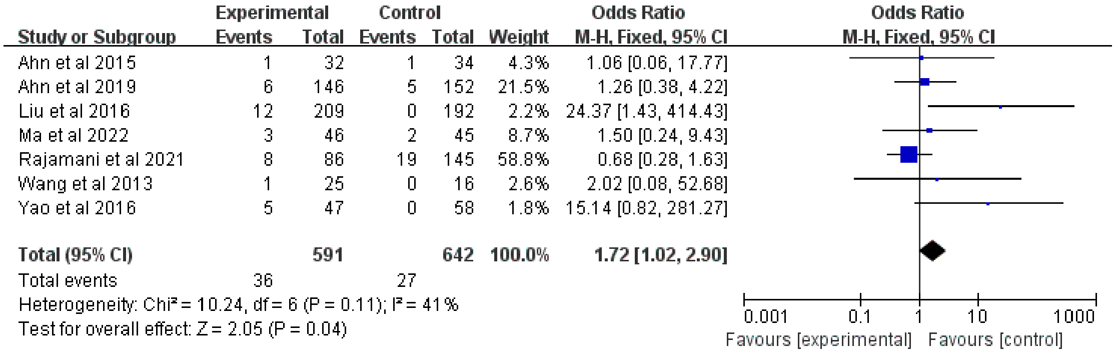

Figure S11b.Meta-analysis for incidence of recurrence between PTED and OD

Study or Subgroup	Experimental Events	Experimental Total	Control Events	Control Total	Weight	Odds Ratio M-H, Fixed, 95% CI
Chen et al 2018	5	80	3	73	6.2%	1.56 [0.36, 6.75]
Chen et al 2020	10	119	5	122	9.5%	2.15 [0.71, 6.48]
Choi et al 2016	2	20	0	23	0.9%	6.35 [0.29, 140.55]
Gibson et al 2016	5	70	2	70	3.9%	2.62 [0.49, 13.96]
Hsu et al 2013	6	57	4	66	7.0%	1.82 [0.49, 6.81]
Jarebi et al 2021	2	29	0	29	1.0%	5.36 [0.25, 116.76]
Kim et al 2007	28	295	38	607	47.3%	1.57 [0.94, 2.61]
Mayer et al1993	3	20	1	20	1.8%	3.35 [0.32, 35.36]
Meyer et al 2020	3	23	3	24	5.4%	1.05 [0.19, 5.83]
Rajamani et al 2021	2	86	11	238	12.0%	0.49 [0.11, 2.26]
Wang et al 2021	5	69	3	86	5.2%	2.16 [0.50, 9.38]
**Total (95% CI)**		**868**		**1358**	**100.0%**	**1.67 [1.17, 2.36]**
Total events	71		70			

Heterogeneity: Chi² = 5.02, df = 10 (P = 0.89); I² = 0%
Test for overall effect: Z = 2.86 (P = 0.004)

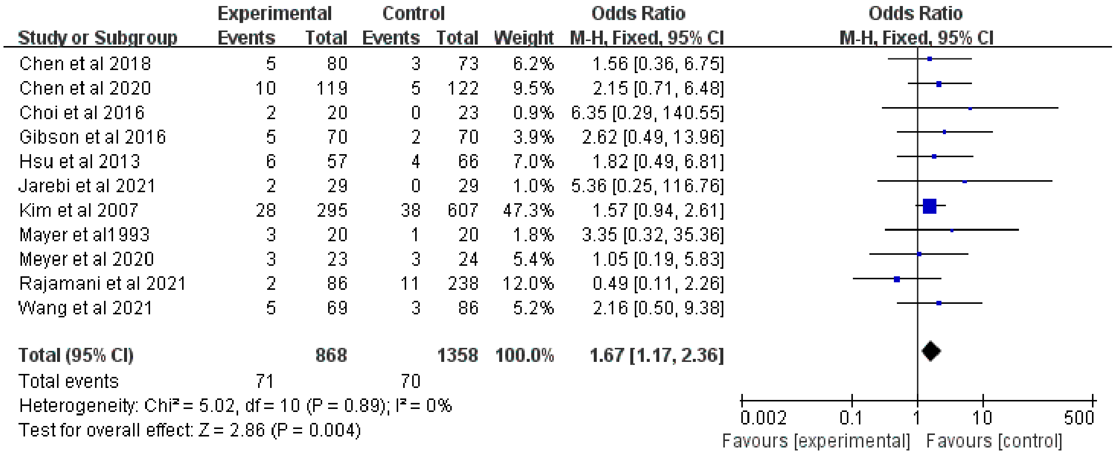

Figure S12a.Meta-analysis for revision rate between PTED and MED

Study or Subgroup	Experimental Events	Experimental Total	Control Events	Control Total	Weight	Odds Ratio M-H, Fixed, 95% CI
Ahn et al 2019	6	146	5	152	30.7%	1.26 [0.38, 4.22]
Kim et al 2019	14	173	59	1683	66.1%	2.42 [1.32, 4.44]
Ma et al 2022	2	46	0	45	3.1%	5.11 [0.24, 109.51]
Rajamani et al 2021	2	86	15	145	0.0%	0.21 [0.05, 0.93]
**Total (95% CI)**		**365**		**1880**	**100.0%**	**2.15 [1.25, 3.70]**
Total events	22		64			

Heterogeneity: Chi² = 1.21, df = 2 (P = 0.55); I² = 0%
Test for overall effect: Z = 2.76 (P = 0.006)

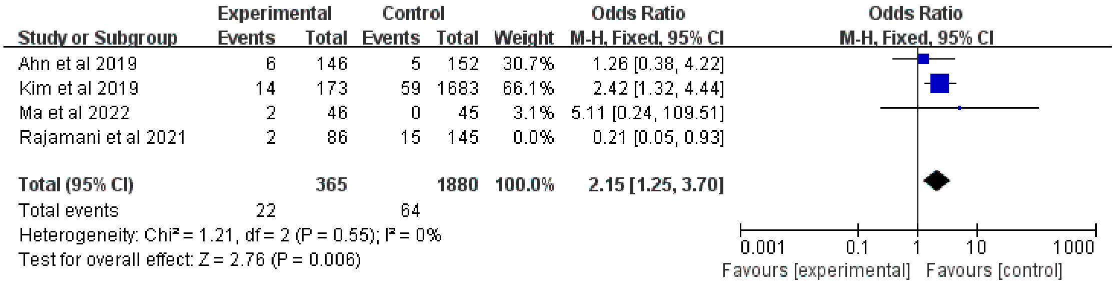

Figure S12b.Meta-analysis for revision rate between PTED and OD

Study or Subgroup	Experimental Events	Experimental Total	Control Events	Control Total	Weight	Odds Ratio M-H, Fixed, 95% CI
Song et al 2017	28	30	27	30	4.1%	1.56 [0.24, 10.05]
Kim et al 2007	260	295	516	607	91.0%	1.31 [0.86, 1.99]
Ariun et al 2015	34	36	46	50	4.9%	1.48 [0.26, 8.54]
**Total (95% CI)**		**361**		**687**	**100.0%**	**1.33 [0.89, 1.98]**
Total events	322		589			

Heterogeneity: Chi² = 0.05, df = 2 (P = 0.98); I² = 0%
Test for overall effect: Z = 1.40 (P = 0.16)

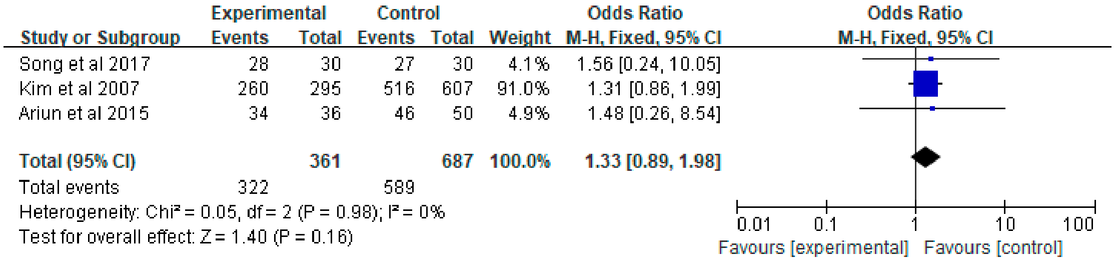

Figure S13a.Meta-analysis for excellent rate of operation between PTED and MED

Study or Subgroup	Experimental Events	Experimental Total	Control Events	Control Total	Weight	Odds Ratio M-H, Fixed, 95% CI
Ding et al 2017	45	50	45	50	13.7%	1.00 [0.27, 3.69]
Jeoug et al 2006	19	22	20	25	7.8%	1.58 [0.33, 7.56]
Li et al 2015	26	30	23	26	10.0%	0.85 [0.17, 4.19]
Liu et al 2016	191	209	183	192	50.1%	0.52 [0.23, 1.19]
Ma et al 2022	40	46	39	45	15.7%	1.03 [0.30, 3.46]
Pan et al 2014	9	10	9	10	2.7%	1.00 [0.05, 18.57]
**Total (95% CI)**		**367**		**348**	**100.0%**	**0.79 [0.48, 1.33]**
Total events	330		319			

Heterogeneity: Chi² = 2.06, df = 5 (P = 0.84); I² = 0%
Test for overall effect: Z = 0.88 (P = 0.38)

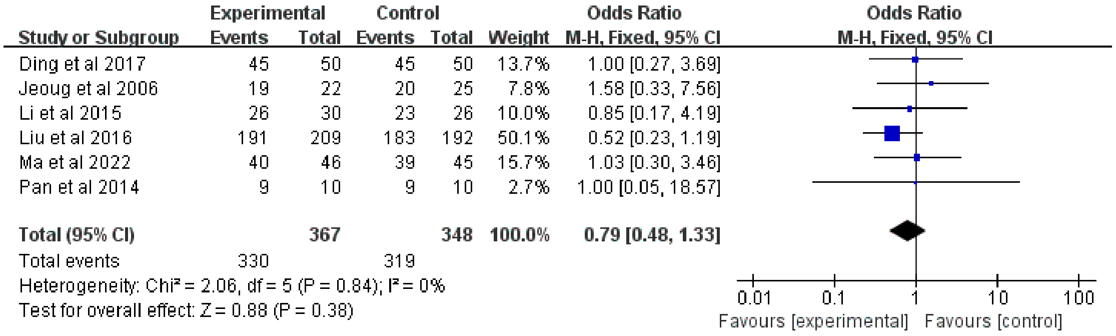

Figure S13b.Meta-analysis for excellent rate of operation between PTED and OD

Supplementary Figure S2 Publication bias

1.PTED VS MED（A-H）：

A

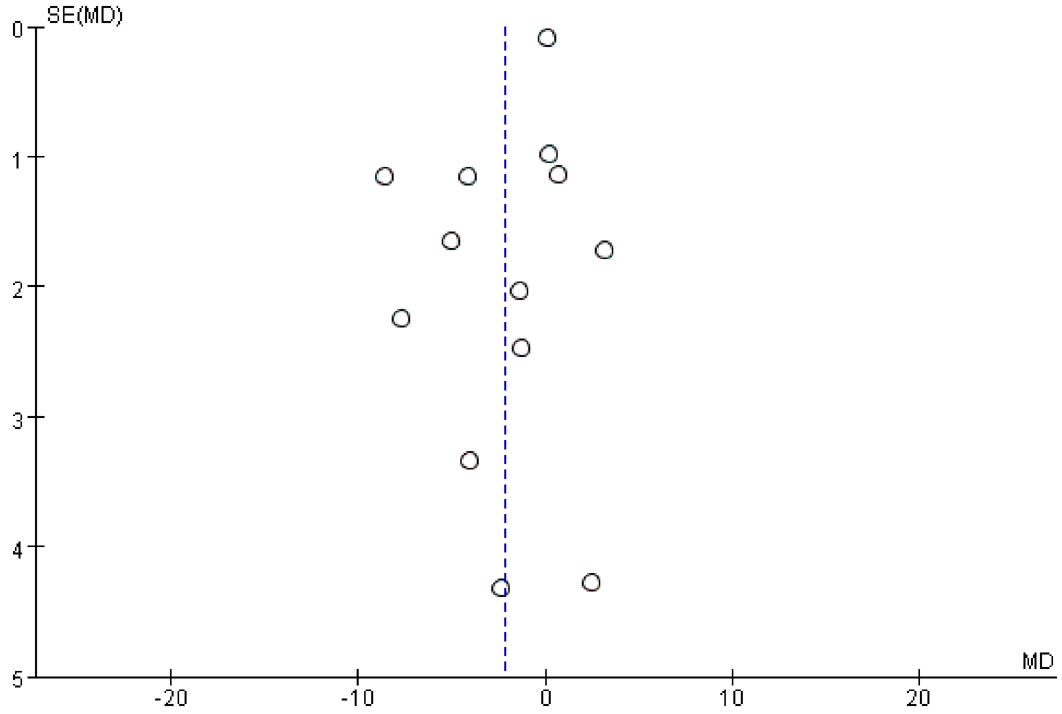

B

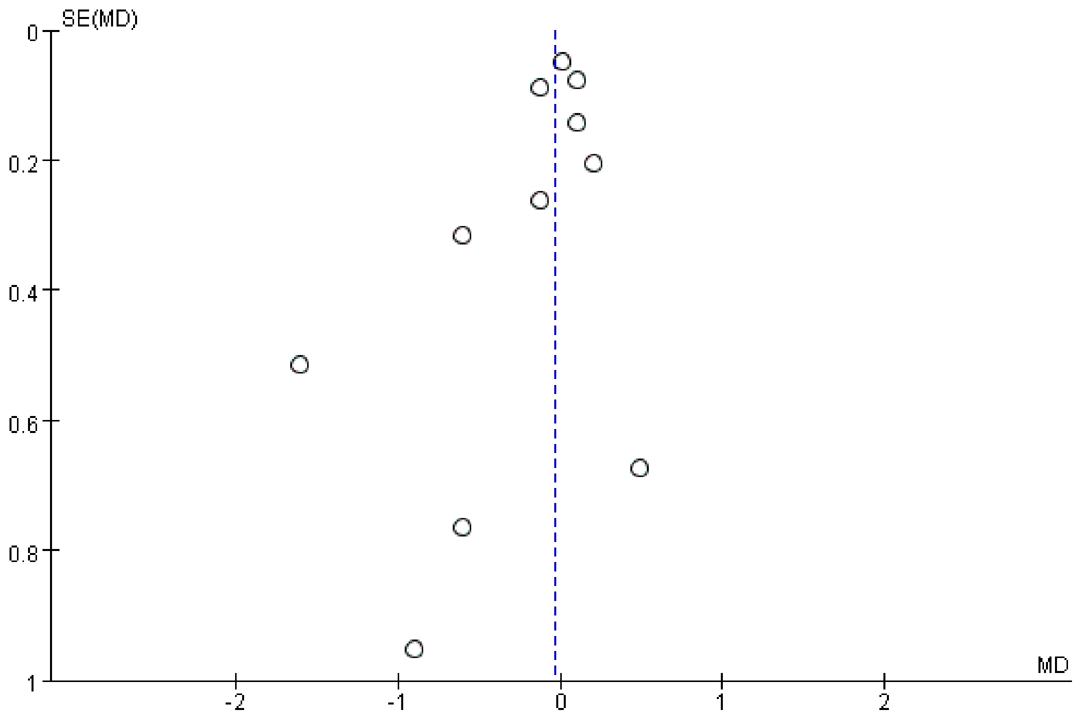

C

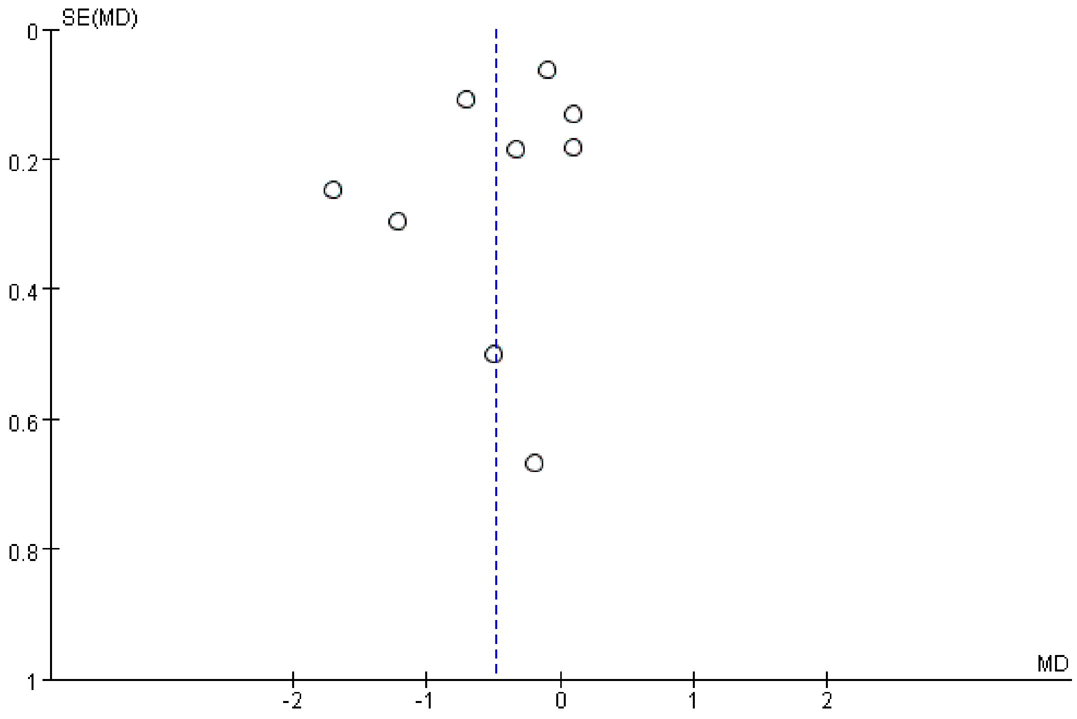

D

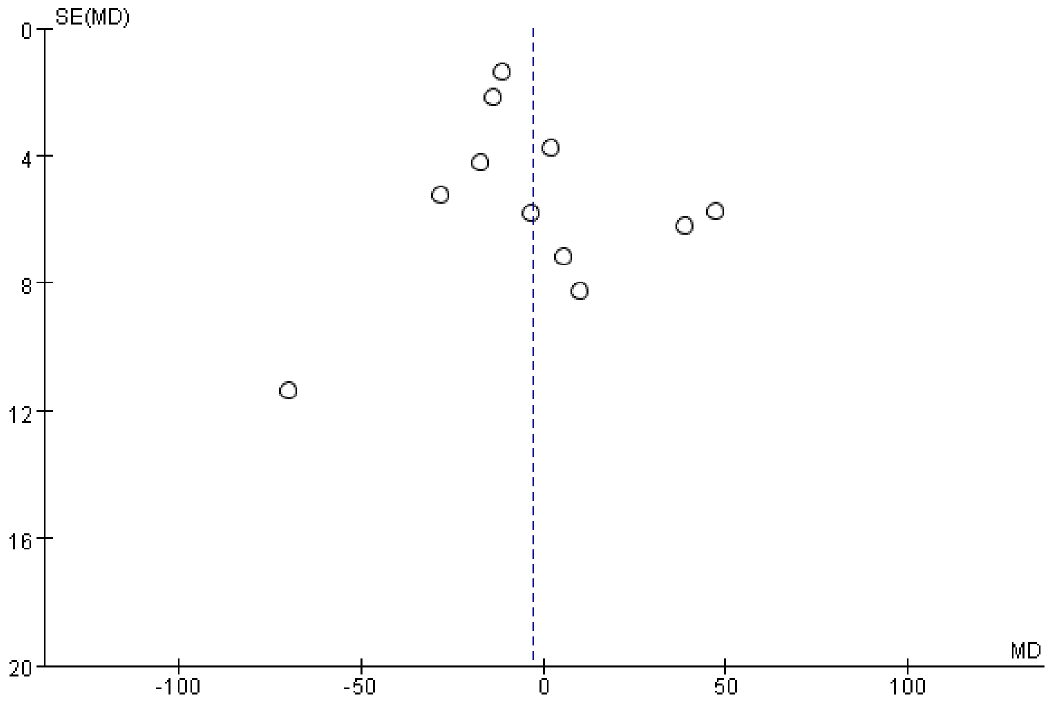

SE(MD)
0
4
8
12
16
20
-100
-50
0
50
100
MD

E

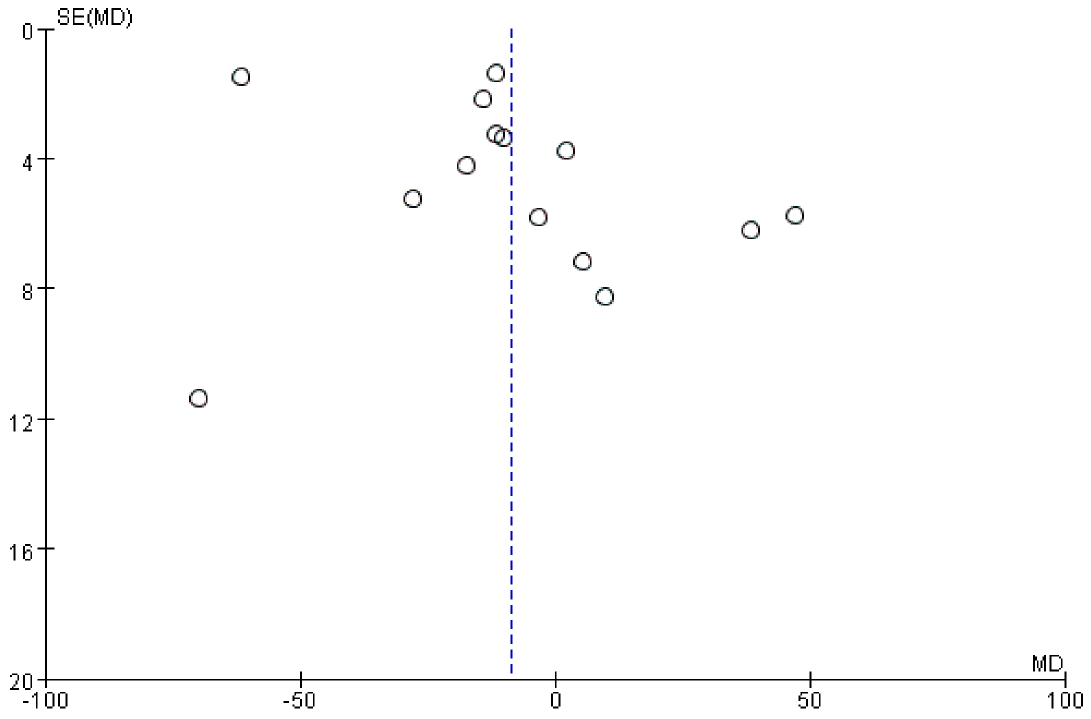

SE(MD)
0
4
8
12
16
20
-100
-50
0
50
100
MD

F

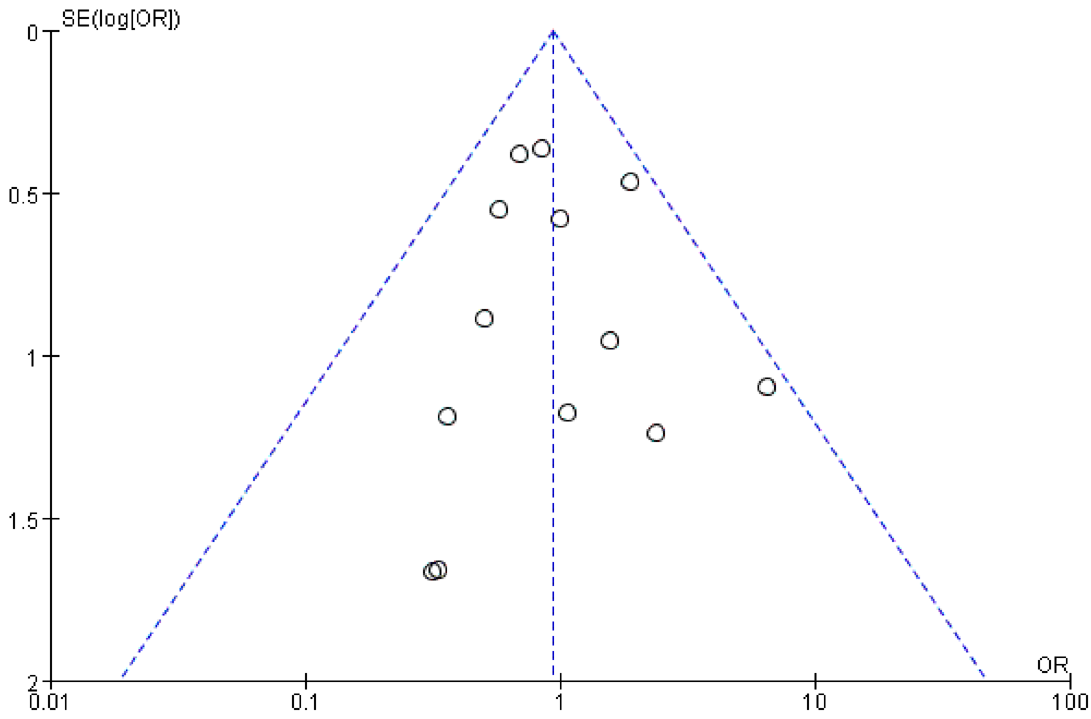
SE(log[OR])
0
0.5
1
1.5
2
OR
0.01
0.1
1
10
100

G

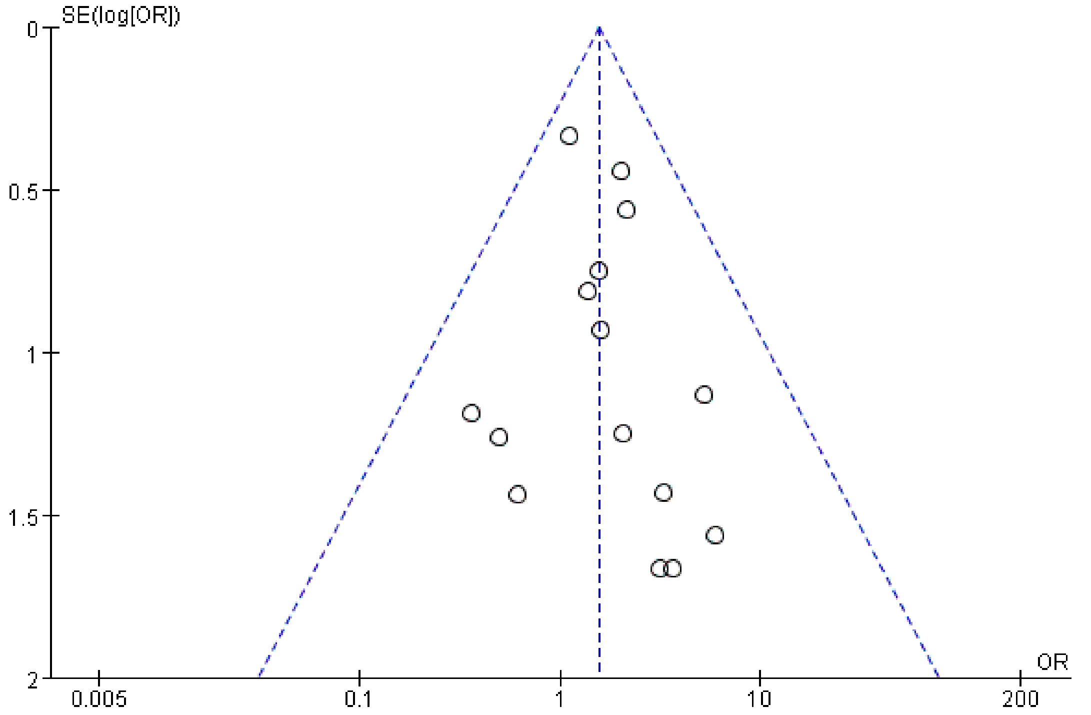
SE(log[OR])
0
0.5
1
1.5
2
OR
0.005
0.1
1
10
200

H

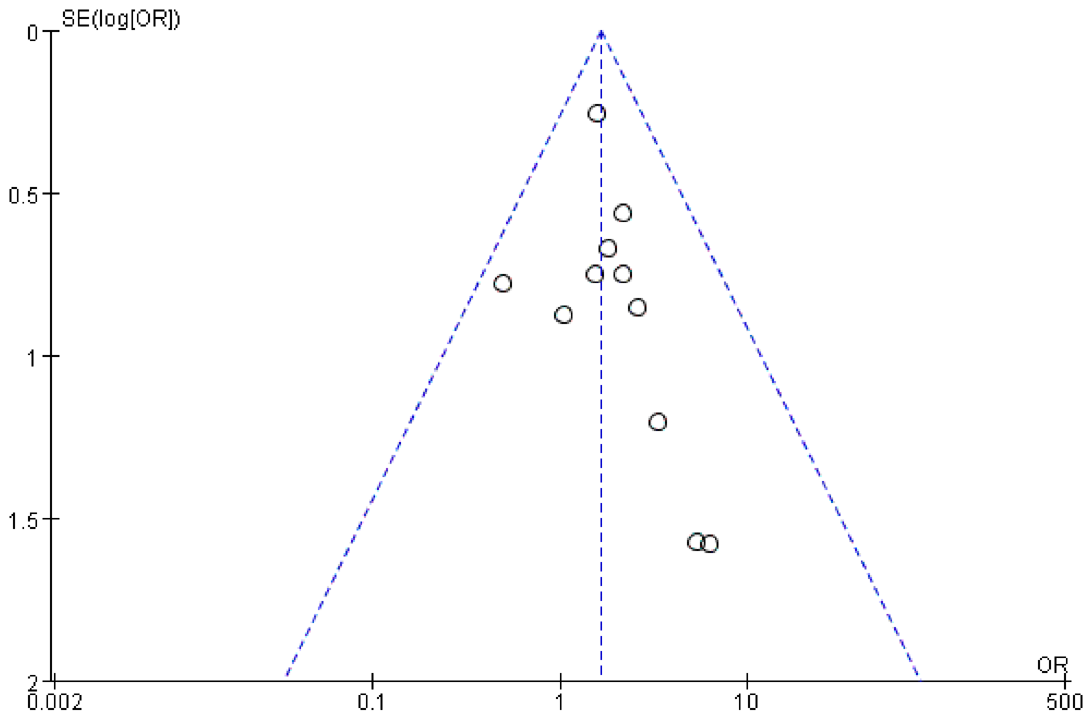

A：ODI index B：VAS leg pain score C：VAS waist/low back pain score D：The average operation time E：Average hospitalization time F：The incidence of postoperative complications G：Postoperative recurrence and residual rate H： Rate of revision surgery

2. PTED VS OD (A-I):

A

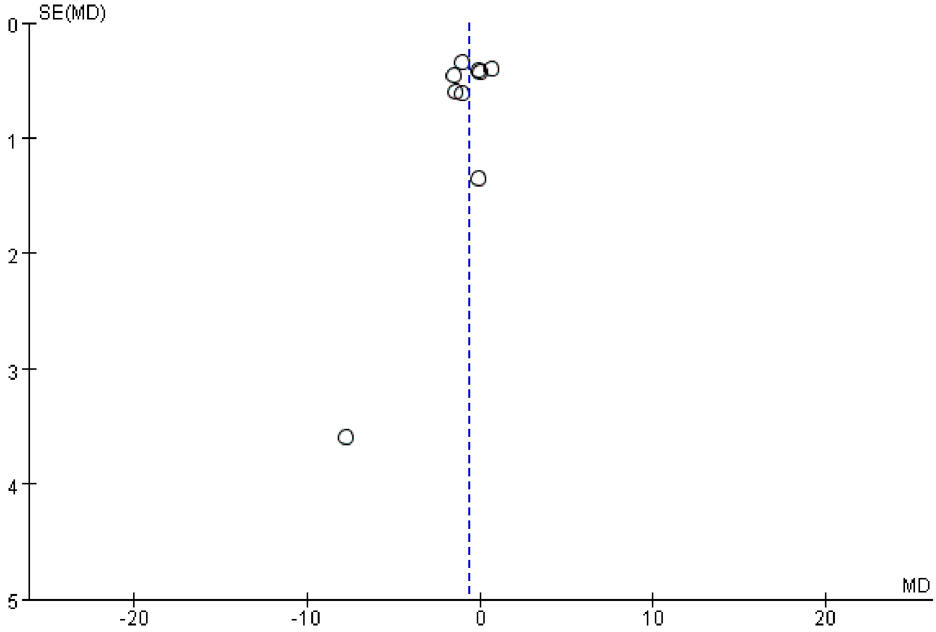

B

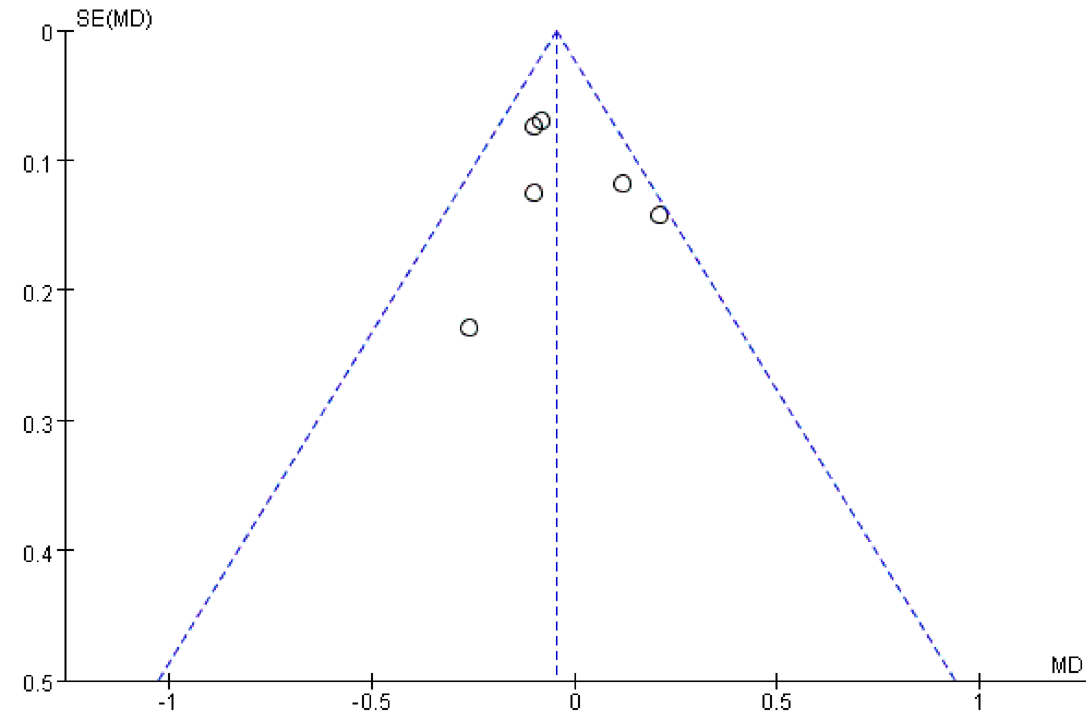

C

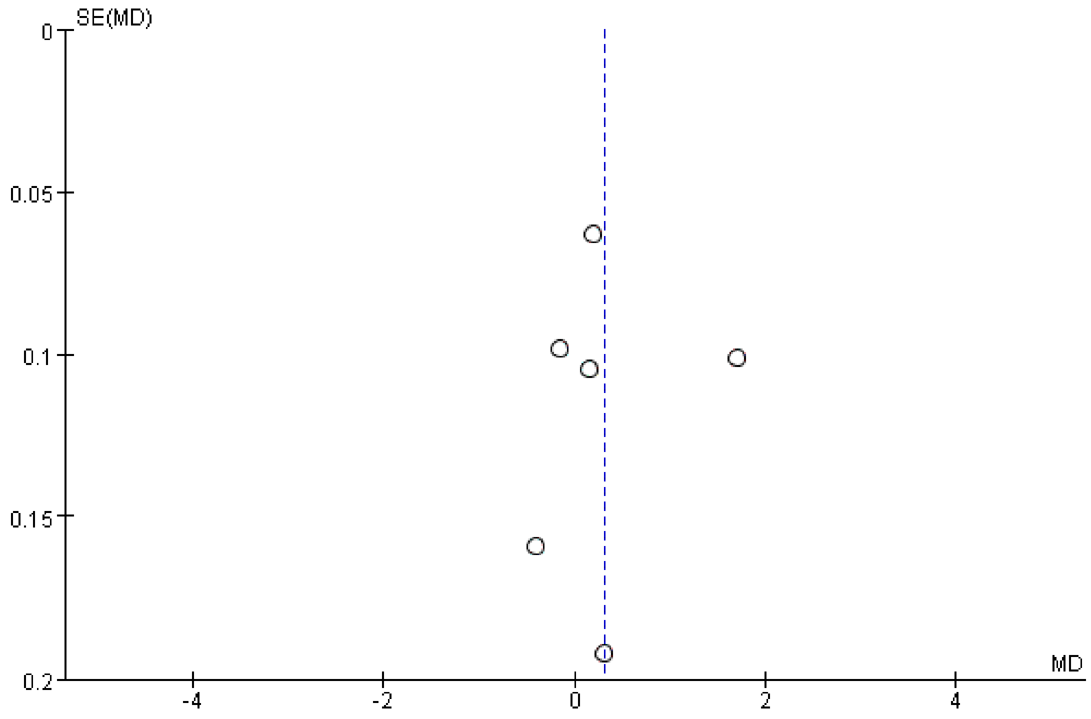

D

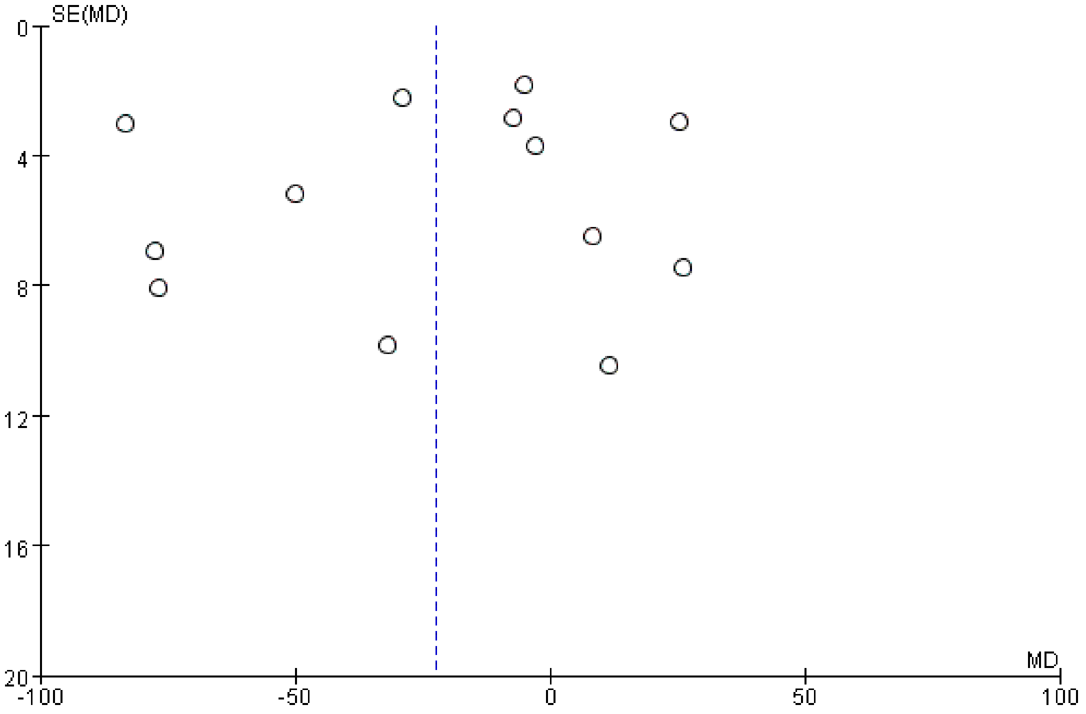
SE(MD)
0
4
8
12
16
20
-100
-50
0
50
100
MD

E

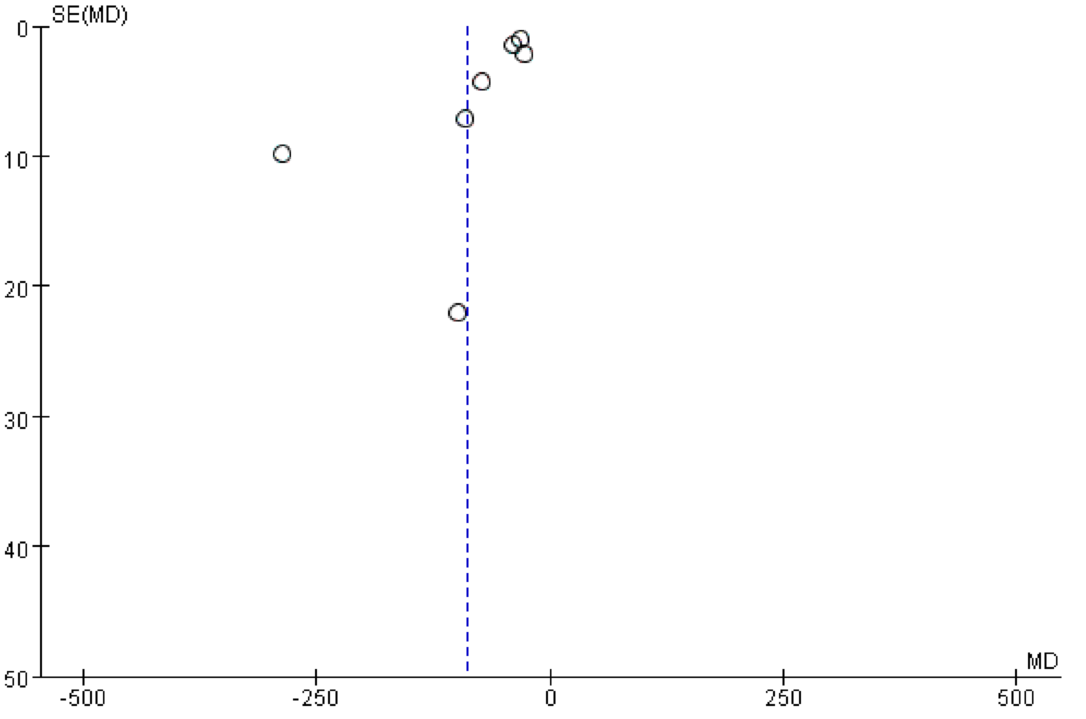
SE(MD)
0
10
20
30
40
50
-500
-250
0
250
500
MD

F

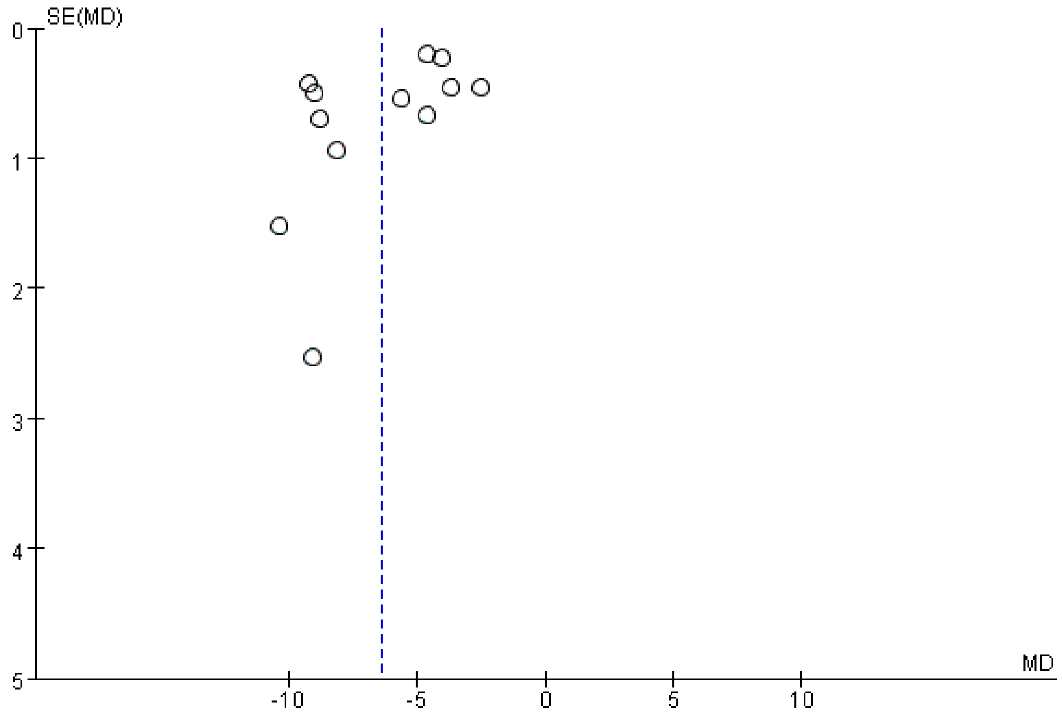

G

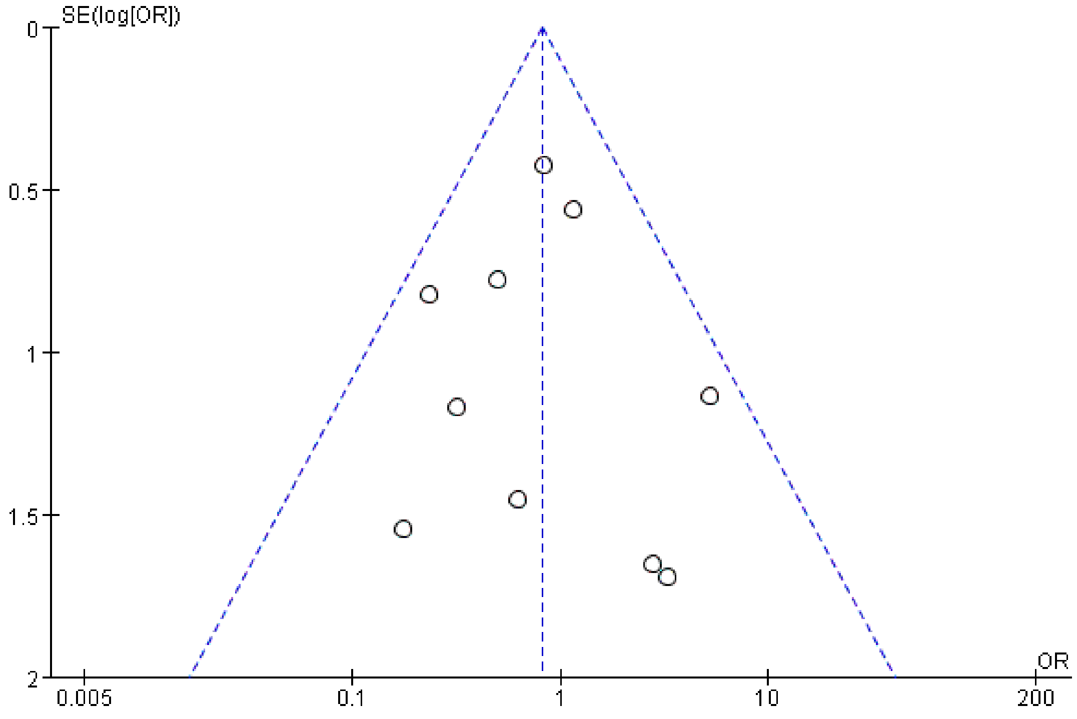

H

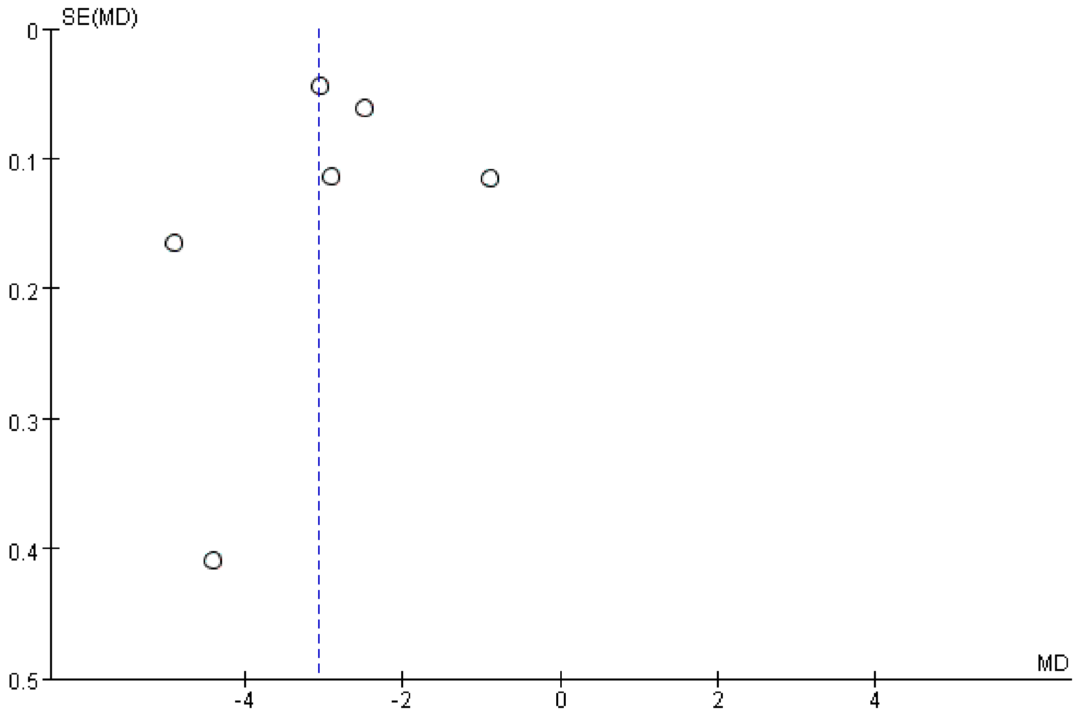

I

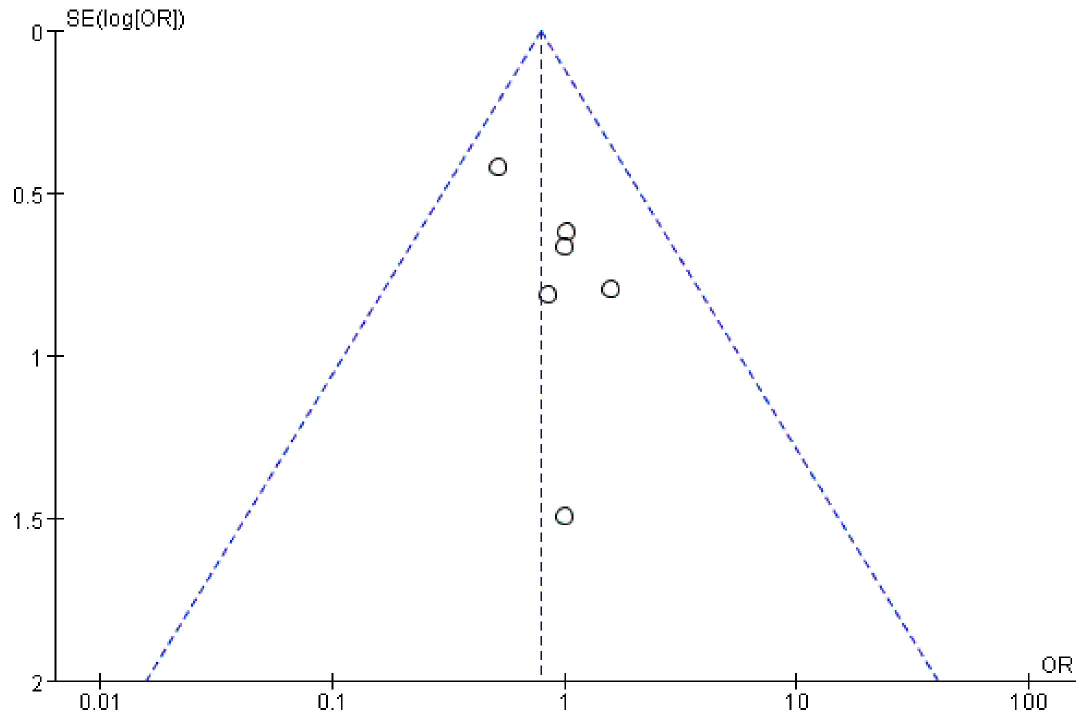

A：ODI index B：VAS leg pain score C: comprehensive VAS score D：The average operation time E：Intraoperatie blood loss F：Average hospitalization time G: The incidence of postoperative complications H：size of the incision I: rates of successful operation
